# Glycolysis-derived alanine from glia fuels neuronal mitochondria for memory in *Drosophila*

**DOI:** 10.1038/s42255-023-00910-y

**Published:** 2023-11-06

**Authors:** Yasmine Rabah, Raquel Francés, Julia Minatchy, Laura Guédon, Coraline Desnous, Pierre-Yves Plaçais, Thomas Preat

**Affiliations:** Energy & Memory, Brain Plasticity Unit, CNRS, ESPCI Paris, PSL Research University, Paris, France

**Keywords:** Classical conditioning, Glial biology, Metabolism

## Abstract

Glucose is the primary source of energy for the brain; however, it remains controversial whether, upon neuronal activation, glucose is primarily used by neurons for ATP production or if it is partially oxidized in astrocytes, as proposed by the astrocyte–neuron lactate shuttle model for glutamatergic neurons. Thus, an in vivo picture of glucose metabolism during cognitive processes is missing. Here, we uncover in *Drosophila* *melanogaster* a glia-to-neuron alanine transfer involving alanine aminotransferase that sustains memory formation. Following associative conditioning, glycolysis in glial cells produces alanine, which is back-converted into pyruvate in cholinergic neurons of the olfactory memory center to uphold their increased mitochondrial needs. Alanine, as a mediator of glia–neuron coupling, could be an alternative to lactate in cholinergic systems. In parallel, a dedicated glial glucose transporter imports glucose specifically for long-term memory, by directly transferring it to neurons for use by the pentose phosphate pathway. Our results demonstrate in vivo the compartmentalization of glucose metabolism between neurons and glial cells during memory formation.

## Abstract (web summary)

In this study, Rabah et al. investigate glucose usage in the brain, and show how glial cells transfer glycolysis-derived alanine to neurons in a fly model, thus supporting memory formation in cholinergic circuits.

## Main

Glucose is the primary energy metabolite consumed by the brain^1^, for which neuronal electrical activity and neurotransmission are the major energy sinks. In mammals, glucose is transported from the blood circulation into both neurons and neighboring glial cells, particularly astrocytes^2^; however, determining which of the two cell types is the primary site of glucose uptake accompanying increased neuronal activity has long been a matter of controversy^3,4^. The astrocyte–neuron lactate shuttle (ANLS) is one hypothesized synaptic mechanism in which the astrocytes are the main glucose consumers. According to this model, astrocytes transfer glycolysis-derived lactate to neurons upon glutamate neurotransmitter reuptake^5,6^. In turn, lactate can be used by neurons for signaling^7^ or oxidative purposes and energy production. The observations that astrocytes can be net suppliers of lactate and that the glycolysis rate is higher in astrocytes than in neurons are well supported^4^. Several studies have shown that long-term memory (LTM) in particular, relies on lactate transfer from astrocytes to neurons^8–10^; however, other studies continue to challenge the ANLS scheme, as they have shown that glucose is preferentially consumed by neurons upon acute stimulation^11,12^. In support of this opposing model, it was reported that neuronal glycolysis is upregulated for energy production in response to neuronal stimulation^13^. Therefore, although the ANLS model aptly solves how energy supply is tailored to synaptic activity, its relevance in vivo is still disputed^3,14^. Moreover, the precise fate of glucose in upholding neuronal oxidative metabolism in vivo, especially during cognitive functions like memory, remains elusive. Notably, the controversy regarding the compartmentalization of glucose metabolism between neurons and glia has crystallized around the ability of lactate to fuel neuronal oxidative metabolism, leaving alternative metabolic derivations of glial glycolysis largely under-investigated. For instance, the l-amino acid alanine is derived from glial glucose metabolism and can serve as a metabolic substrate^15–17^, making it a potential substitute for lactate as an energy source.

The unmatched genetic, behavioral and cellular-resolution imaging toolsets available to *Drosophila* studies provide a powerful and temporally precise means to investigate, in vivo, the metabolic processes engaged in high-order brain functions such as memory. *Drosophila* can form aversive olfactory memory upon the association between an odorant and electric shocks, and a variety of conditioning protocols have been designed that evoke memories with different properties and persistence^18,19^. All forms of olfactory memories are encoded within specific subsets of neurons in the mushroom body (MB), a bilateral structure of about 2,000 cholinergic neurons in each brain hemisphere^20^. *Drosophila* neurons are surrounded by several types of glial cells, making this model relevant for the study of neuron-glia metabolic coupling supporting memory formation.

Recently, we successfully unveiled several metabolic adaptations in vivo that underlie memory formation, including neuron-glia metabolic coupling processes^21,22^. We found that the early phase of LTM formation is sustained by the rapid upregulation of neuronal pyruvate metabolism by mitochondria^23^. Furthermore, we revealed the existence of a concomitant glucose shuttle from perisomatic glial cells (known as cortex glia) to neuronal somata that supplies the neuronal pentose phosphate pathway (PPP) specifically for LTM formation, such that MB neuron glucose utilization is uncoupled from pyruvate oxidation for LTM^21^. Because the cellular and molecular origins of pyruvate sustaining memory had not yet been determined, this prompted us to identify the mechanisms supporting upregulated pyruvate metabolism for memory formation.

Here, we have uncovered the reliance of specific forms of aversive memories (middle-term memory (MTM) and LTM), on pyruvate metabolism in MB neurons. Our results indicate that a main source of neuronal pyruvate for both MTM and LTM is the amino acid l-alanine. We further show that alanine is produced by neighboring cortex glia through glycolysis. Finally, we revealed that two distinct glucose transporters contribute in parallel to glucose import by glial cells for distinct fates: one for glial glycolysis that sustains MTM and LTM, and one for direct glucose transfer to MB neurons specifically for LTM. Overall, our results demonstrate the in vivo compartmentalization of glucose metabolism between neurons and glial cells for memory formation, with alanine transfer from glia to neurons bridging glial glycolysis with neuronal mitochondrial pyruvate consumption.

## Results

### Mitochondrial pyruvate uptake sustains specific memory forms

Following a single pairing of electric shocks with an odorant (single-cycle training), associative odor-avoidance memory persists for several hours but decays within 1 d. Based on genetic and circuit analyses, this memory was dissected into two distinct components, designated as MTM^18,19^ and middle-term anesthesia-resistant memory (MT-ARM). A stable protein synthesis-dependent LTM, which persists for several days, can be formed by subjecting flies to repeated conditioning cycles separated by rest intervals (5× spaced training)^18,19^. MTM and LTM are encoded in the same subset of MB neurons and retrieved through the same downstream circuits^24,25^, so that LTM formation is thought to rely on initial cellular substrates that are shared with MTM. Notably, repeated conditioning cycles without rest (5× massed training) induce a distinct form of consolidated memory (LT-ARM). This memory differs from LTM in terms of persistence^18^, the underlying neuronal circuits^25^ and support genes^26,27^ and metabolic regulations^21,23^.

**Fig. 1:**
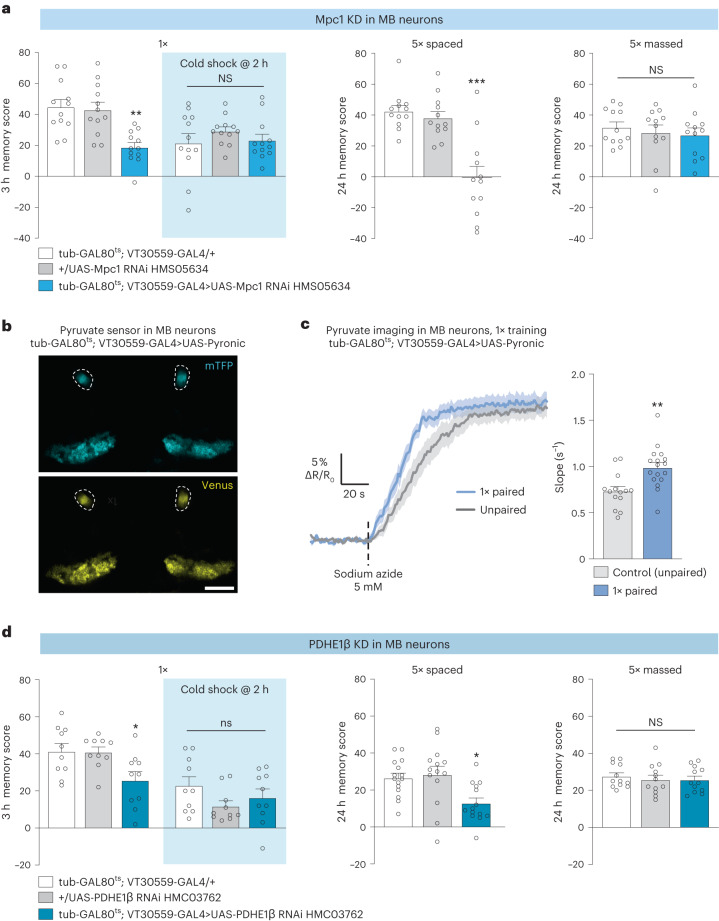
Pyruvate consumption by MB neurons increases upon memory formation. **a**, *Mpc1* knockdown (KD) in adult MB neurons impaired memory after single-cycle (1×) training (*n* = 12, *F*_2,33_ = 11.40, *P* = 0.00017) and spaced training (*n* = 12, *F*_2,33_ = 19.36, *P* = 3.10^−6^), but did not affect memory after single-cycle training followed by cold shock (*n* = 12, *F*_2,33_ = 0.83, *P* = 0.45) or massed training (*n* = 12, *F*_2,33_ = 0.36, *P* = 0.70). **b**, The pyruvate sensor Pyronic was expressed in adult MB neurons. The two images show the mTFP and Venus channels. The dashed lines delimit bundles of MB neuron axons, namely the vertical lobes, where the pyruvate FRET signal was quantified. Scale bar, 50 µm (Supplementary Video 1). **c**, Single-cycle training elicited a faster pyruvate accumulation in MB neuron axons following sodium azide application (5 mM) compared to non-associative unpaired training (*n* = 14 (control); *n* = 17 (1×), *t*_29_ = 3.39, *P* = 0.002). **d**, *PDHE1β* knockdown in adult MB neurons impaired memory after single-cycle training (*n* = 10, *F*_2,27_ = 5.08, *P* = 0.01) and spaced training (*n* = 14 (blue and gray bars); *n* = 15 (white bar), *F*_2,40_ = 6.25, *P* = 0.004), but did not affect memory after single-cycle training followed by cold shock (*n* = 10, *F*_2,27_ = 1.91, *P* = 0.17) or massed training (*n* = 12, *F*_2,33_ = 0.27, *P* = 0.76). All data are presented as mean ± s.e.m. Asterisks (*P < 0.05; **P < 0.01; ***P < 0.001; NS, not significant, *P* > 0.05) illustrate the significance level of a two-sided *t*-test or of the least significant pairwise comparison following one-way analysis of variance (ANOVA). Source data

**Extended Data Fig. 1:**
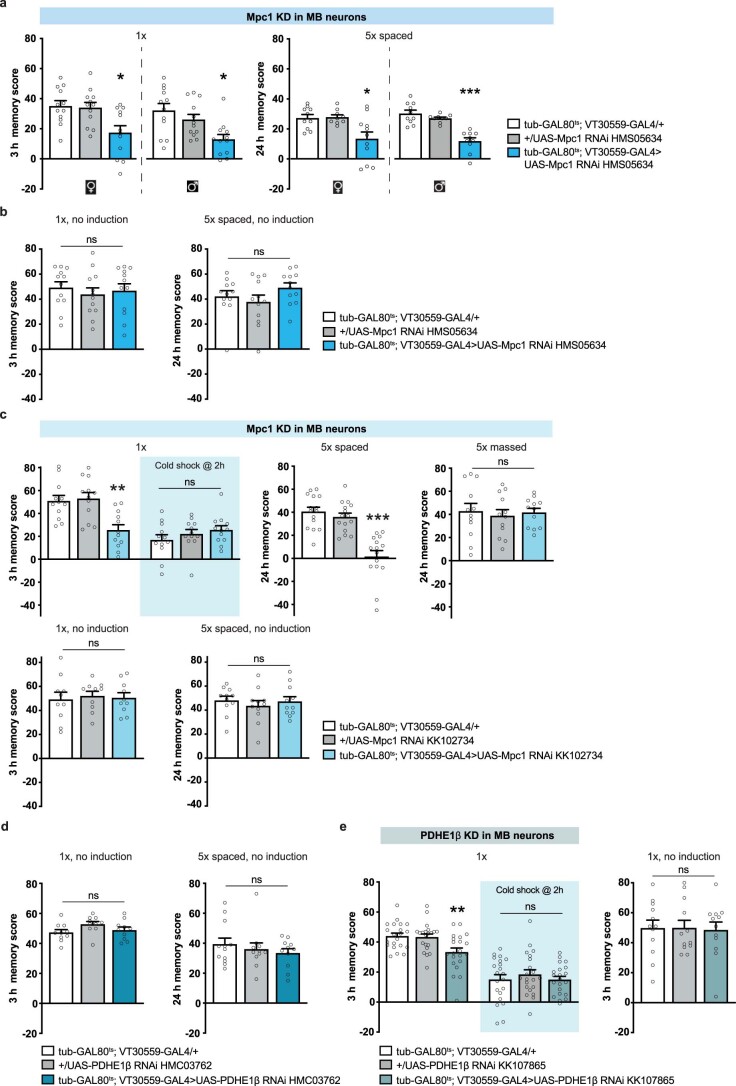
Behavioral controls of *Mpc1* and *PDHE1ß* KD in MB neurons (related to Fig. 1). **a**. *Mpc1* knockdown (KD) in MB neurons impaired memory after single-cycle training in both females and males (females: n = 12, F_2,33_ = 6.46, P = 0.004; males: n = 12, F_2,33_ = 6.81, P = 0.003). *Mpc1* knockdown (KD) in MB neurons impaired memory after spaced training in both females and males (females: n = 9-11, F_2,27_ = 6.45, P = 0.005; males: n = 9-10, F_2,26_ = 25.98, P = 6.10^−7^). **b**. Without induction of *Mpc1* RNAi, memory after single-cycle (1x) training (n = 12, F_2,33_ = 0.28, P = 0.76) and spaced training (n = 12, F_2,33_ = 1.53, P = 0.23) was normal. **c**. The behavioral experiments were performed with a second RNAi against *Mpc1*. *Mpc1* KD in adult MB neurons impaired memory after single-cycle training (n = 12, F_2,33_ = 9.74, P = 0.00047) and spaced training (n = 15, F_2,42_ = 26.03, P = 4.10^−8^), but did not affect memory after single-cycle training followed by cold shock (n = 12, F_2,33_ = 1.14, P = 0.33) or massed training (n = 12, F_2,33_ = 0.14, P = 0.87). Without induction of *Mpc1* RNAi, memory after single-cycle training (n = 10, F_2,27_ = 0.09, P = 0.91) and spaced training (n = 11, F_2,30_ = 0.37, P = 0.69) was normal. **d**. Without induction of *PDHE1β* RNAi, memory after single-cycle training (n = 10, F_2,27_ = 2.12, P = 0.14) and spaced training (n = 11 (blue and gray bars);n = 12 (white bar), F_2,31_ = 0.60, P = 0.55) was normal. **e**. The behavioral experiments were performed with a second RNAi against *PDHE1β*. *PDHE1β* knockdown in adult MB neurons impaired memory after single-cycle training (n = 21, F_2,60_ = 7.05, P = 0.002), but did not affect memory after single-cycle training followed by cold shock (n = 21, F_2,60_ = 0.48, P = 0.62). Without induction of *PDHE1β* RNAi, memory after single-cycle training (n = 12, F_2,33_ = 0.02, P = 0.98) was normal. All data are presented as mean ± SEM. Asterisks (*P < 0.05; **P < 0.01; ***P < 0.001; ns: not significant, P > 0.05) illustrate the significance level of two-sided t-test, or of the least significant pairwise comparison following 1-way ANOVA. Source data

Pyruvate import into mitochondria via the mitochondrial pyruvate carrier Mpc1 (ref. ^28^) is an obligatory step in the oxidative metabolism of glucose derivatives. To probe the reliance of *Drosophila* memory formation on neuronal pyruvate mitochondrial metabolism, we investigated the effect of impaired pyruvate uptake on memory using RNAi interference (RNAi) targeted against *Mpc1* expression. For this, an *Mpc1* RNAi was expressed selectively in MB neurons using the VT30559-GAL4 driver^23^. To avoid putative developmental issues, the thermosensitive GAL4 inhibitor GAL80^ts^ was ubiquitously coexpressed (tub-GAL80^ts^(ref. ^29^); Methods provides details) so that GAL4-mediated RNAi expression would be induced only at the adult stage by heat activation for 3 d before conditioning. After single-cycle training, 3-h memory was strongly but partially impaired (Fig. 1a and Extended Data Fig. 1a). Cold-shock treatment, which erases the labile MTM component while leaving the MT-ARM component intact, revealed that this impairment corresponds to a complete loss of MTM (Fig. 1a). LTM, measured 24 h after spaced training, was also fully impaired by *Mpc1* knockdown (Fig. 1a). By contrast, LT-ARM, measured 24 h after massed training, was not affected by Mpc1 knockdown (Fig. 1a). This is in agreement with our previous report that LTM formation relies on the pyruvate dehydrogenase (PDH) mitochondrial enzyme complex and involves increased mitochondrial pyruvate consumption in MB neurons^23^. MTM and LTM performances were normal when RNAi expression was not induced (Extended Data Fig. 1a), which rules out the possibility that the observed memory defects could be due to leaky RNAi expression during development. We also verified that innate sensory acuity for the relevant stimuli was normal in the flies of interest (Supplementary Table 1). These behavioral experiments were replicated using a second, non-overlapping *Mpc1* RNAi (Extended Data Fig. 1b). MTM and LTM were not impaired when *Mpc1* RNAi was not induced (Extended Data Fig. 1c). Together, these results demonstrate that MTM and LTM formation rely critically on mitochondrial pyruvate import into MB neurons.

Using in vivo pyruvate imaging experiments to monitor the pyruvate consumption by mitochondria in MB neurons, we previously reported that LTM relies on an increased mitochondrial pyruvate flux in MB vertical lobes (a structure encompassing the axonal projections of MB neurons). This increased pyruvate metabolism occurred in the first 3 h after the end of spaced training^23^. As the behavioral results following *Mpc1* knockdown also suggest a role for pyruvate metabolism in MTM, we performed similar imaging experiments after single-cycle training. For this, we expressed the pyruvate FRET sensor Pyronic^30^ in MB neurons (Fig. 1b) and measured pyruvate flux in MB vertical lobes within 1.5 h after training. Pyruvate accumulation was measured following the application of sodium azide, a blocker of mitochondrial complex IV, as in our previously characterized method^23^. Here, we observed a faster accumulation of pyruvate in single-cycle trained flies as compared to flies that had a non-associative (unpaired) conditioning protocol, as revealed by the increased slope (Fig. 1c). This result shows that an increased mitochondrial pyruvate consumption by MB neurons occurs after single-cycle training and spaced training, in the first hours following training.

To confirm that pyruvate is actually routed to the mitochondrial tricarboxylic acid (TCA) cycle after single-cycle training, we targeted one of the enzymes of the pyruvate dehydrogenase (PDH) complex, which catalyzes the conversion of pyruvate to acetyl-CoA. Knockdown of the β subunit of *PDHE1* in adult MB neurons impaired MTM (Fig. 1d and Extended Data Fig. 1d) and, as previously reported using another RNAi, LTM formation (Fig. 1d)^23^. MTM and LTM were not impaired when *PDHE1β* RNAi was not induced (Extended Data Fig. 1d). LT-ARM was not affected by *PDHE1β* knockdown (Fig. 1d). Furthermore, sensory acuity for the relevant stimuli was normal in the flies of interest (Supplementary Table 1). All together, these results show that MB neuron mitochondrial pyruvate consumption is increased for both MTM and LTM.

### Pyruvate is produced from alanine in the MB for memory

**Fig. 2:**
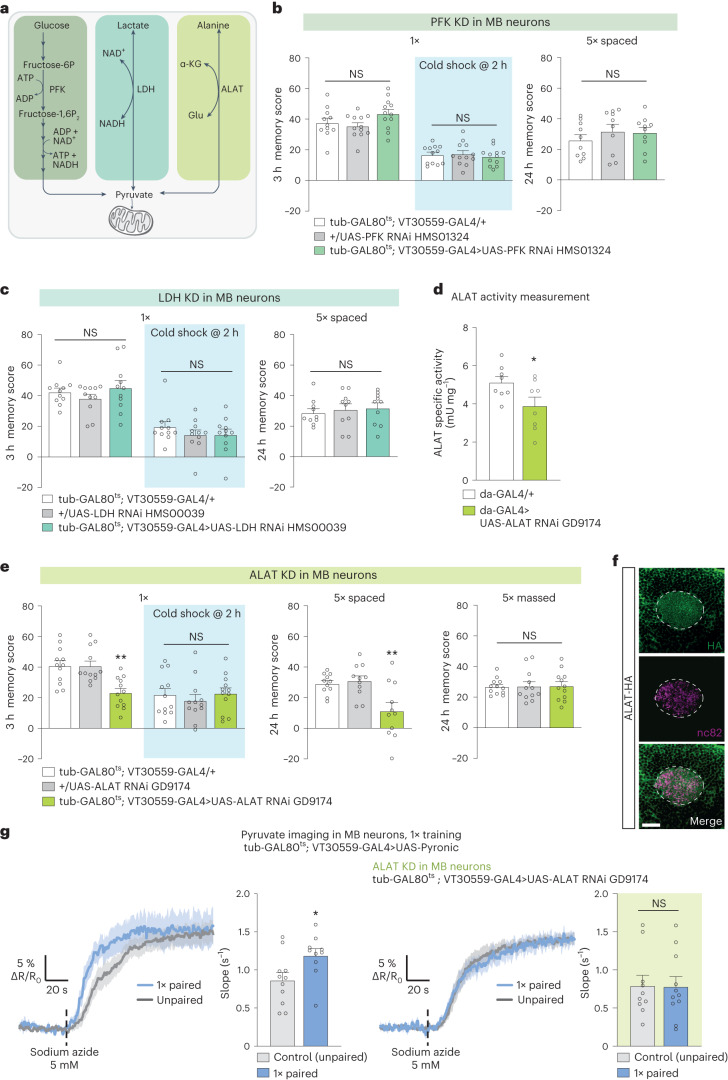
Pyruvate is produced from alanine for MTM and LTM. **a**, Scheme of the three main pyruvate production routes. **b**, *PFK* KD in adult MB neurons did not affect memory after single-cycle training (*n* = 12 for all datasets except the tub-GAL80^ts^; VT30559-GAL4/+ control without cold shock (*n* = 11), *F*_2,32_ = 2.43, *P* = 0.10; after cold shock: *n* = 12, *F*_2,33_ = 21, *P* = 0.81) or spaced training (*n* = 10, *F*_2,32_ = 0.65, *P* = 0.53). **c**, *LDH* knockdown in adult MB neurons did not affect memory after single-cycle training (*n* = 11, *F*_2,30_ = 0.98, *P* = 0.39; after cold shock, *n* = 11, *F*_2,30_ = 0.75, *P* = 0.48) or after spaced training (*n* = 10, F_2,27_ = 0.25, *P* = 0.78). **d**, ALAT enzymatic activity in heads was decreased due to ubiquitous KD of *ALAT*, *n* = 8, *t*_14_ = 2.23, *P* = 0.04). **e**, *ALAT* KD in adult MB neurons impaired memory after single-cycle training (*n* = 12, *F*_2,33_ = 10.2, *P* = 0.00035) and spaced training (*n* = 11, *F*_2,30_ = 7.95, *P* = 0.002), but did not affect memory after single-cycle training followed by cold shock (*n* = 12, *F*_2,33_ = 0.41, *P* = 0.66) or massed training (*n* = 12, *F*_2,33_ = 0.012, *P* = 0.99). **f**, Immunohistochemistry of *ALAT-HA* brain (green) with pan-neuronal counterstaining (nc82, red) in the MB calyx region (dashed line). Scale bar, 20 µm. **g**, Single-cycle training elicited a faster pyruvate accumulation in MB neuron axons following sodium azide application (5 mM) compared to non-associative unpaired training (*n* = 11 (control); *n* = 10 (1×), *t*_19_ = 2.39, *P* = 0.027). This effect was impaired by *ALAT* KD in adult MB neurons (*n* = 10 (control); *n* = 11 (1×), *t*_19_ = 0.064, *P* = 0.95). All data are presented as mean ± s.e.m. Asterisks (*P < 0.05; **P < 0.01; NS, not significant, *P* > 0.05) illustrate the significance level of a two-sided *t*-test or of the least significant pairwise comparison following one-way or two-way ANOVA. Source data

**Extended Data Fig. 2:**
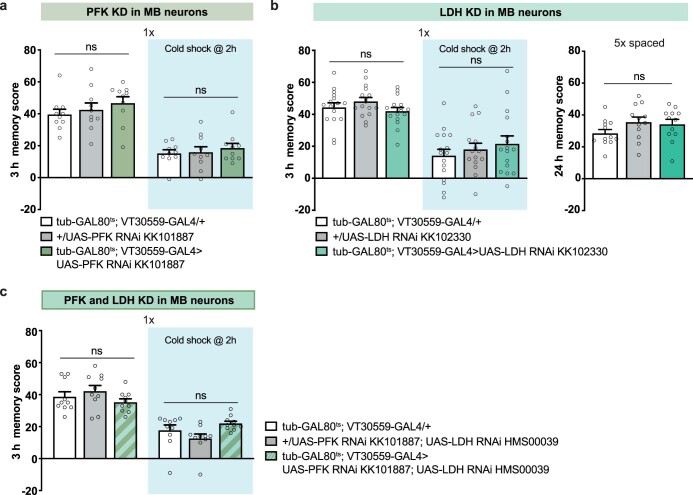
Behavioral controls of *PFK* and *LDH* KD in MB neurons (related to Fig. 2). **a**. *PFK* knockdown (KD) in adult MB neurons using a second RNAi did not affect memory after single-cycle training (n = 10, F_2,27_ = 0.85, P = 0.44) or single-cycle training followed by cold shock (n = 10, F_2,27_ = 0.35, P = 0.71). **b**. *LDH* knockdown in adult MB neurons using a second RNAi did not affect memory after single-cycle training (n = 15-16, F_2,45_ = 1.52, P = 0.23), single-cycle training followed by cold shock (n = 16, F_2,44_ = 0.76, P = 0.47), or spaced training (n = 12, F_2,33_ = 1.73, P = 0.19). **c**. Double KD of *PFK* and *LDH* in MB neurons did not affect memory after single-cycle training (n = 10, F_2,27_ = 1.37, P = 0.27) or after single-cycle training followed by cold shock (n = 10, F_2,27_ = 3.19, P = 0.06) All data are presented as mean ± SEM. Asterisks (*P < 0.05; **P < 0.01; ***P < 0.001; ns: not significant, P > 0.05) illustrate the significance level of two-sided t-test, or of the least significant pairwise comparison following 1-way ANOVA. Source data

**Extended Data Fig. 3:**
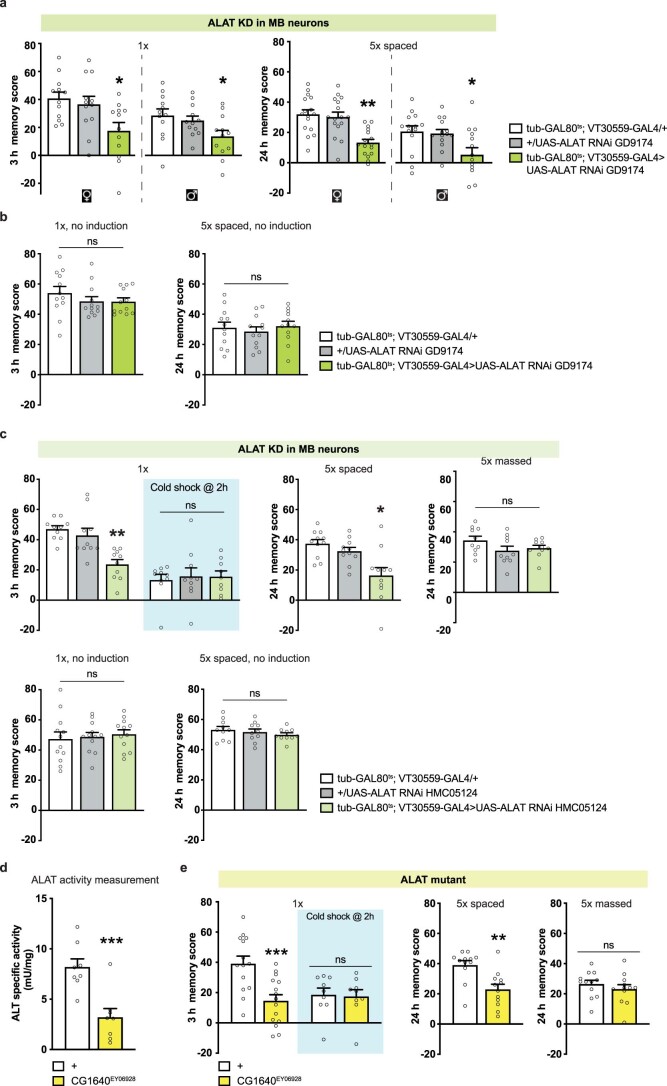
Behavioral controls of *ALAT* KD in MB neurons (related to Fig. 2). **a**. *ALAT* knockdown (KD) in MB neurons impaired memory after single-cycle training in both females and males (females: n = 12, F_2,33_ = 5.13, P = 0.01; males: n = 12, F_2,33_ = 3.50, P = 0.04). *ALAT* KD in MB neurons impaired memory after spaced training in both females and males (females: n = 16, F_2,45_ = 13.14, P = 3.10^−5^; males: n = 14, F_2,39_ = 5.08, P = 0.01). **b**. Without induction of *ALAT* RNAi in MB neurons, memory after single-cycle training (n = 12, F_2,33_ = 0.92, P = 0.41) and spaced training (n = 12, F_2,33_ = 0.32, P = 0.73) was normal. **c**. The behavioral experiments were performed with a second RNAi against *ALAT*. *ALAT* knockdown in adult MB neurons impaired memory after single-cycle training (n = 10, F_2,27_ = 12.61, P = 0.00014) and spaced training (n = 11, F_2,30_ = 8.76, P = 0.001), but did not affect memory after single-cycle training followed by cold shock (n = 10, F_2,27_ = 0.09, P = 0.91) or massed training (n = 10, F_2,27_ = 1.90, P = 0.17). Without induction of *ALAT* RNAi in MB neurons, memory after single-cycle training (n = 12, F_2,33_ = 0.19, P = 0.83) and spaced training (n = 12, F_2,27_ = 0.77, P = 0.47) was normal. **d**. ALAT enzymatic activity in heads is decreased in flies mutant for *ALAT* (n = 8, t_14_ = 4.19, P = 0.0009). **e**. The *ALAT* mutation impaired memory after single-cycle training (n = 14 (control); n = 15 (mutant), t_27_ = 3.86, P = 0.0006) and spaced training (n = 12, t_22_ = 3.56, P = 0.002), but did not affect memory after single-cycle training followed by cold shock (n = 9, t_16_ = 0.18, P = 0.86) or massed training (n = 12, t_22_ = 0.86, P = 0.40). All data are presented as mean ± SEM. Asterisks (*P < 0.05; **P < 0.01; ***P < 0.001; ns: not significant, P > 0.05) illustrate the significance level of two-sided t-test, or of the least significant pairwise comparison following 1-way ANOVA. Source data

Next, we aimed to identify the source of neuronal pyruvate for MTM and LTM. The main routes of pyruvate production are (1) glycolysis, which allows pyruvate production from glucose through a sequence of ten enzymatic reactions; (2) lactate conversion into pyruvate, catalyzed by lactate dehydrogenase (LDH); and (3) l-alanine transamination into pyruvate, catalyzed by alanine aminotransferase (ALAT)^31^ (Fig. 2a). We therefore tested each of these three routes for a role in memory formation in MB neurons. In adult MB neurons, knockdown of phosphofructokinase (PFK), encoding an enzyme that catalyzes a rate-limiting step in glycolysis, had no effect on MTM or LTM (Fig. 2b and Extended Data Fig. 2a)^21^. Knockdown of *LDH* in adult MB neurons had no effect on MTM or LTM (Fig. 2c and Extended Data Fig. 2b). To rule out the possibility that knockdown of glycolysis and lactate dehydrogenation were compensated by each other, we knocked down both *PFK* and *LDH* in adult MB neurons. As this resulted in intact MTM (Extended Data Fig. 2c), we focused on the third route of pyruvate production, through the transamination of l-alanine catalyzed by ALAT. In *Drosophila*, the gene *CG1640* encodes a putative ALAT (http://flybase.org/reports/FBgn0030478). Ubiquitous knockdown of *CG1640* decreased ALAT activity in *Drosophila* heads (Fig. 2d), confirming that the product of *CG1640* features ALAT enzymatic activity. We therefore refer to *CG1640* as *ALAT*. Knocking down *ALAT* in adult MB neurons led to an impairment of both MTM and LTM (Fig. 2e and Extended Data Fig. 3a). MTM and LTM were not impaired when *ALAT* RNAi was not induced (Extended Data Fig. 3b). Memory after massed training was not affected by *ALAT* knockdown (Fig. 2e). Sensory acuity for the relevant stimuli was normal in the flies of interest (Supplementary Table 2). We replicated these behavioral experiments using a second, non-overlapping *ALAT* RNAi (Extended Data Fig. 3c). MTM and LTM defects were observed in *ALAT* mutant flies (CG1640^EY06928^), which also showed impaired ALAT enzymatic activity (Extended Data Fig. 3d,e). To verify that ALAT is indeed expressed in MB neurons, we generated an *ALAT-HA* knock-in line. Immunohistochemistry experiments on ALAT-HA brains showed immunoreactivity against the HA-tag in MB neurons, and in particular in the calyx which corresponds to the dendritic field, where it colocalized with neuronal marker nc82 (Fig. 2f). These results confirmed the expression of *ALAT* in MB neurons.

To confirm that alanine is indeed a source of pyruvate for MTM, we performed in vivo pyruvate imaging in MB vertical lobes while knocking down *ALAT* in adult MB neurons. Single-cycle training failed to elicit an increased pyruvate accumulation when *ALAT* was knocked down in adult MB neurons (Fig. 2g). Overall, these results show that MTM and LTM do not rely on neuronal glycolysis or lactate dehydrogenation, but instead rely on alanine metabolism as a relevant source of pyruvate in MB neurons for memory formation.

### Cortex glia transfers glycolysis-derived alanine to neurons

**Fig. 3:**
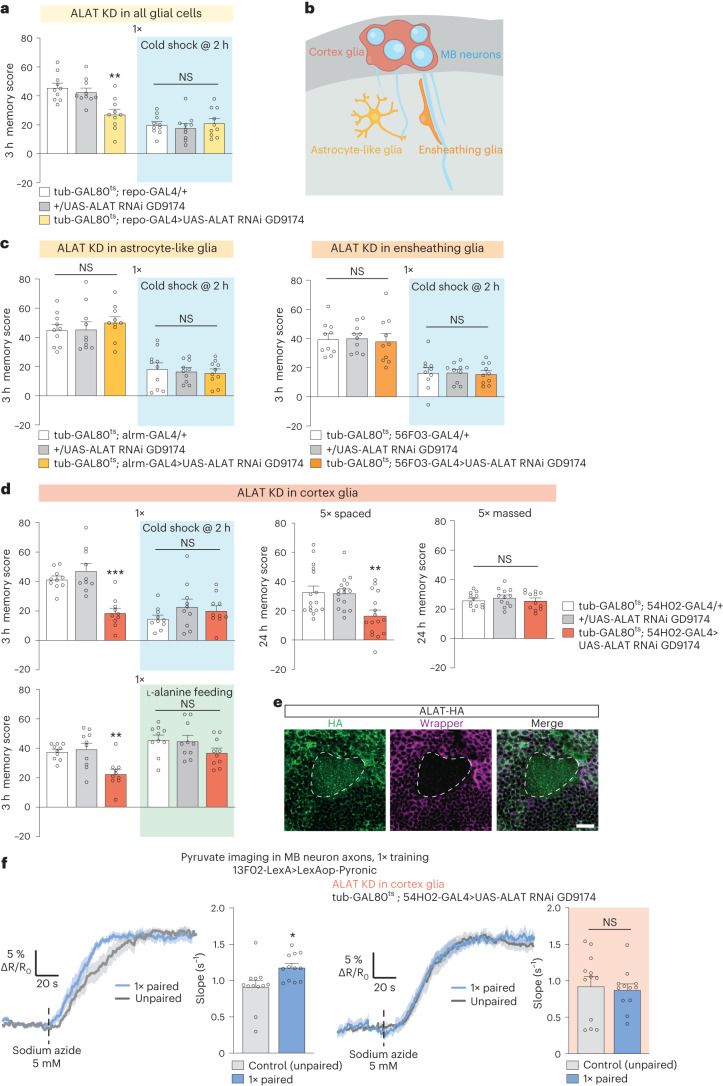
Cortex glia transfer alanine to MB neurons for MTM and LTM. **a**, *ALAT* KD in adult glia impaired memory after single-cycle training (*n* = 10, *F*_2,27_ = 10.56, *P* = 0.0004), but did not affect memory after single-cycle training followed by cold shock (*n* = 10, *F*_2,27_ = 0.38, *P* = 0.69). **b**, Subtypes and localization of *Drosophila* glia contacting neurons in the brain. **c**, *ALAT* KD in adult astrocyte-like glia (left) or ensheathing glia (right) did not affect memory after single-cycle training (astrocyte-like glia, *n* = 10, *F*_2,27_ = 0.48, *P* = 0.62; ensheathing glia, *n* = 10, *F*_2,27_ = 0.07, *P* = 0.93) or single-cycle training followed by cold shock (astrocyte-like glia, *n* = 10, *F*_2,27_ = 0.18, *P* = 0.83; ensheathing glia, *n* = 10, *F*_2,27_ = 0.037, *P* = 0.96). **d**, *ALAT* KD in adult cortex glia impaired memory after single-cycle training (*n* = 10, *F*_2,27_ = 18.67, *P* = 8.10^−6^) and spaced training (*n* = 16, *F*_2,45_ = 6.57, *P* = 0.0031), but did not affect memory after single-cycle training followed by cold shock (*n* = 10, *F*_2,27_ = 1.26, *P* = 0.30) or massed training (*n* = 12, *F*_2,33_ = 0.45, *P* = 0.64). l-alanine feeding (60 mM) for 72 h before training rescued memory defects induced by *ALAT* KD in cortex glia (two-way ANOVA, *n* = 10; genotype effect, *F*_1,54_ = 12.69, *P* = 0.0008; food medium effect, *F*_2,54_ = 1.04, *P* = 0.36; interaction, *F*_2,54_ = 9.60, *P* = 0.0003). **e**, Immunohistochemistry of *ALAT-HA* brain (green) with cortex glia counterstaining (wrapper, red-dashed line, MB calyx region) Scale bar, 20 µm. **f**, The increased mitochondrial pyruvate flux elicited by single-cycle training (*n* = 12, *t*_22_ = 2.65, *P* = 0.015) was impaired by *ALAT* knockdown in adult cortex glia (*n* = 12, *t*_22_ = 0.34, *P* = 0.74). All data are presented as mean ± s.e.m. Asterisks (*P < 0.05; **P < 0.01; ***P < 0.001; NS, not significant, *P* > 0.05) illustrate the significance level of a two-sided *t*-test or of the least significant pairwise comparison following one-way ANOVA. Source data

**Extended Data Fig. 4:**
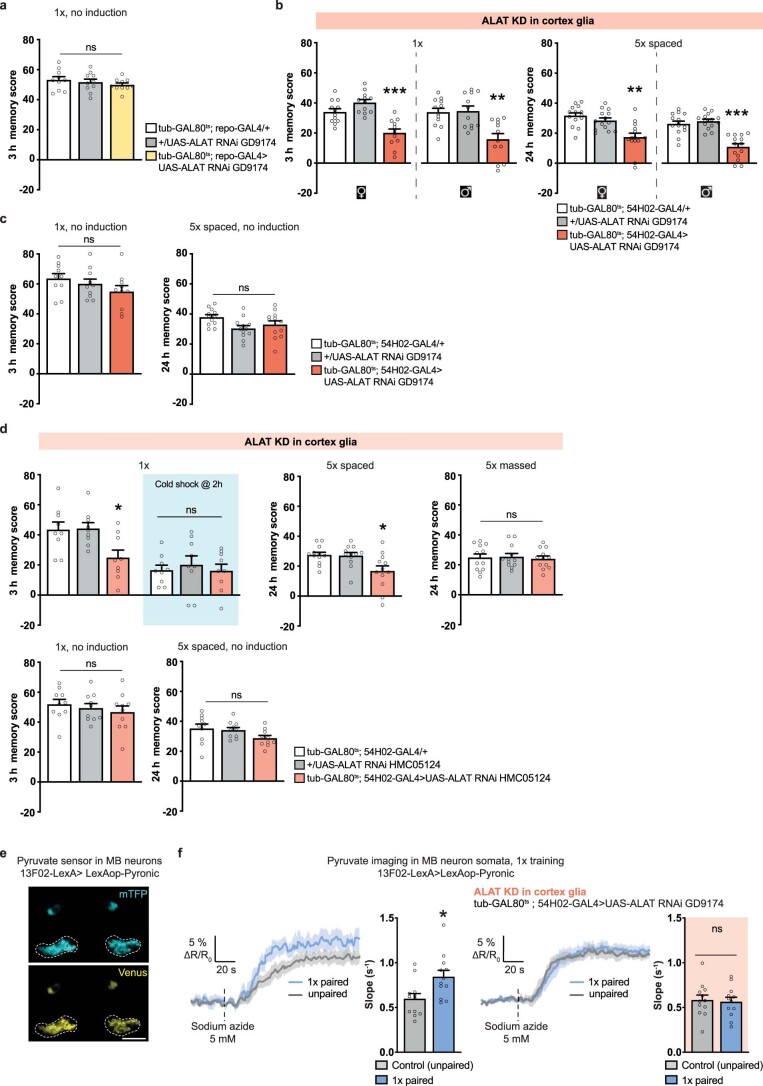
Behavioral controls of *ALAT* KD in glia, and pyruvate imaging in *ALAT* KD (related to Fig. 3). **a**. Without induction of *ALAT* RNAi in all glial cells, memory after single-cycle training (n = 10, F_2,27_ = 0.77, P = 0.47) was normal. **b**. *ALAT* knockdown (KD) in cortex glia impaired memory after single-cycle training in both females and males (females: n = 12, F_2,33_ = 20.68, P = 1.10^−6^; males: n = 12, F_2,33_ = 10.68, P = 3. 10^−4^). *ALAT* KD in cortex glia impaired memory after spaced training in both females and males (females: n = 14, F_2,39_ = 12.48, P = 6.10^−5^; males: n = 14, F_2,39_ = 26.99, P = 4.10^−8^). **c**. Without induction of *ALAT* RNAi in cortex glia, memory after single-cycle training (n = 10, F_2,27_ = 1.57, P = 0.23) and spaced training (n = 11, F_2,33_ = 2.43, P = 0.10) was normal. **d**. The behavioral experiments were performed with a second RNAi against *ALAT*. *ALAT* KD in adult cortex glia impaired memory after single-cycle training (n = 9, F_2,24_ = 5.46, P = 0.01) and spaced training (n = 12, F_2,33_ = 5.60, P = 0.008), but did not affect memory after single-cycle training followed by cold shock (n = 10, F_2,27_ = 0.22, P = 0.80) or massed training (n = 12, F_2,33_ = 0.10, P = 0.90). Without induction of *ALAT* RNAi in cortex glia, memory after single-cycle training (n = 10, F_2,27_ = 0.56, P = 0.58) and spaced training (n = 10, F_2,27_ = 2.34, P = 0.12) was normal. **e**. The pyruvate sensor Pyronic was expressed in adult MB neurons. The dashed lines delimit the somata of MB neurons where the pyruvate FRET signal was quantified. Scale bar: 50 µm. **f**. Single-cycle training elicited a faster pyruvate accumulation in MB neuron somata following sodium azide application (5 mM) as compared to non-associative unpaired training (n = 12, t_22_ = 2.70, P = 0.01). *ALAT* knockdown in adult cortex glia impaired the single-cycle induced increase in pyruvate accumulation in MB neuron somata following sodium azide application (n = 12, t_22_ = 0.27, P = 0.79). All data are presented as mean ± SEM. Asterisks (*P < 0.05; **P < 0.01; ***P < 0.001; ns: not significant, P > 0.05) illustrate the significance level of two-sided t-test, or of the least significant pairwise comparison following 1-way ANOVA. Source data

Previously, it was shown in *Drosophila* that alanine can be produced as a byproduct of glial glycolysis and released by glial cells^15^. We therefore hypothesized that the alanine required for neuronal pyruvate production and consumption for MTM and LTM originates from neighboring glial cells. The transamination reaction catalyzed by ALAT is reversible, so that alanine can be produced from glycolysis-derived pyruvate. ALAT knockdown in all adult glial cells using repo-GAL4 impaired MTM (Fig. 3a) and MTM was not impaired when RNAi was not induced (Extended Data Fig. 4a), suggesting that alanine is produced in glia for MTM. Next, we examined which specific glial cell types require ALAT. The *Drosophila* brain contains several types of glial cells, including three types that are in close contact with different neuronal compartments (Fig. 3b). The ensheathing glia delimit major brain structures, whereas astrocyte-like glial cells contact synapses and cortex glia enwrap neuron somata^32–34^. *ALAT* knockdown in either adult astrocyte-like glia (using the alrm-GAL4 driver) or ensheathing glia (using the 56F03-GAL4 driver) had no effect on MTM (Fig. 3c); however, *ALAT* knockdown in adult cortex glia (using the 54H02-GAL4 driver) impaired both MTM and LTM (Fig. 3d and Extended Data Fig. 4b). MTM and LTM were not impaired when RNAi was not induced (Extended Data Fig. 4c). LT-ARM was not affected by ALAT knockdown (Fig. 3d). Sensory acuity for the relevant stimuli was normal in the flies of interest (Supplementary Table 3). We then replicated these behavioral experiments using a second, non-overlapping *ALAT* RNAi (Extended Data Fig. 4d). Notably, the MTM defect induced by *ALAT* knockdown in adult cortex glia was rescued by exposing flies to an alanine-enriched diet, suggesting that alanine itself is the substrate transferred to MB neurons for MTM (Fig. 3d). To confirm that *ALAT* is expressed in cortex glia, we performed immunohistochemistry experiments on brains from *ALAT-HA* flies and detected HA immunoreactivity in wrapper-positive cortex glia (Fig. 3e).

**Extended Data Fig. 5:**
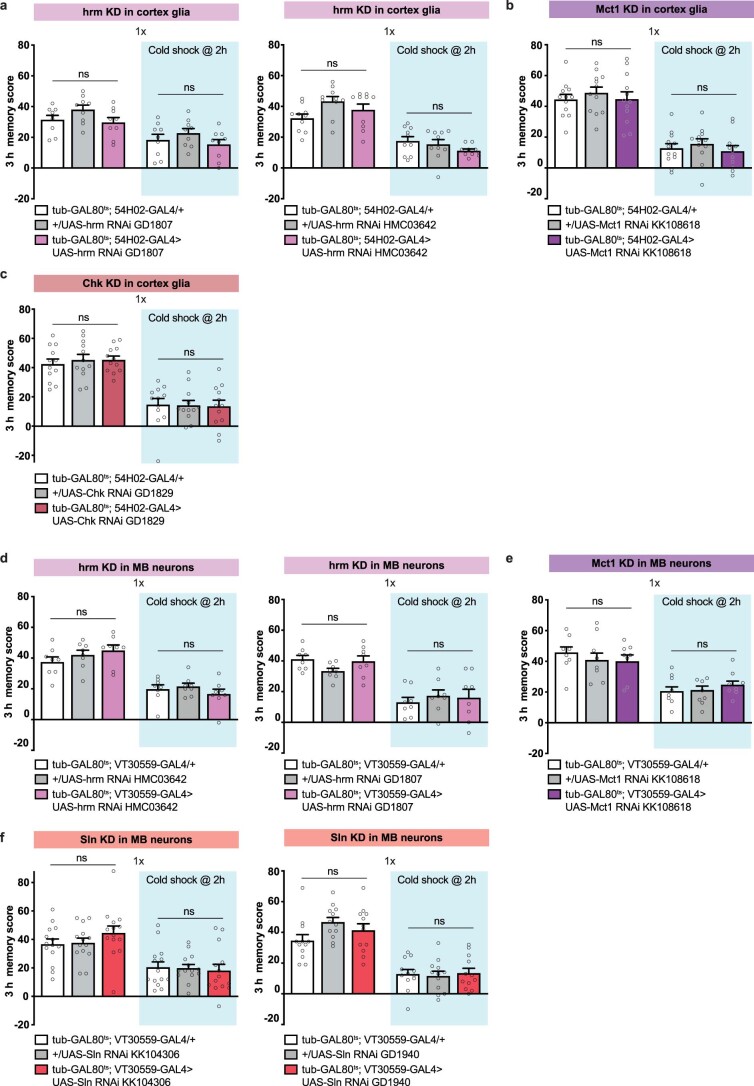
Behavioral controls of *Hrm*, *Mct1*, *Chk* and *Sln* KD in MB neurons (related to Fig. 3). **a**. *Hrm* knockdown (KD) in adult cortex glia did not affect memory after single-cycle training (RNAi GD1807: n = 9, F_2,24_ = 2.13, P = 0.14; RNAi HMC03642: n = 10, F_2,27_ = 2.88, P = 0.07) or single-cycle training followed by cold shock (RNAi GD1807: n = 9, F_2,24_ = 1.25, P = 0.30; RNAi HMC03642: n = 10, F_2,27_ = 1.67, P = 0.21). **b**. *Mct1* KD in adult cortex glia did not affect memory after single-cycle training (n = 12, F_2,33_ = 0.36, P = 0.70) or single-cycle training followed by cold shock (n = 12, F_2,33_ = 0.49, P = 0.62). **c**. *Chaski* KD in adult cortex glia did not affect memory after single-cycle training (n = 12, F_2,33_ = 0.25, P = 0.78) or single-cycle training followed by cold shock (n = 12, F_2,33_ = 0.02, P = 0.98). **d**. *Hrm* KD in adult MB neurons did not affect memory after single-cycle training (RNAi GD1807: n = 8, F_2,21_ = 2.31, P = 0.12; RNAi HMC03642: n = 8, F_2,21_ = 1.30, P = 0.29) or single-cycle training followed by cold shock (RNAi GD1807: n = 8, F_2,21_ = 0.25, P = 0.78; RNAi HMC03642: n = 8, F_2,21_ = 0.79, P = 0.47). **e**. *Mct1* KD in adult MB neurons did not affect memory after single-cycle training (n = 8, F_2,21_ = 0.23, P = 0.79) or single-cycle training followed by cold shock (n = 8, F_2,21_ = 0.86, P = 0.44). **f**. *Sln* KD in adult MB neurons did not affect memory after single-cycle training (RNAi KK104306: n = 14, F_2,39_ = 1.16, P = 0.32; RNAi GD1940: n = 12, F_2,33_ = 2.56, P = 0.09) or single-cycle training followed by cold shock (RNAi KK104306: n = 14, F_2,39_ = 0.11, P = 0.89; RNAi GD1940: n = 12, F_2,33_ = 0.09, P = 0.91). All data are presented as mean ± SEM. Asterisks (*P < 0.05; **P < 0.01; ***P < 0.001; ns: not significant, P > 0.05) illustrate the significance level of the least significant pairwise comparison following 1-way ANOVA. Source data

We hypothesized that cortex glia alanine is transferred to MB neurons as a pyruvate precursor. If so, the loss of ALAT activity solely in adult cortex glia should prevent the increased pyruvate consumption in MB neurons after training. Indeed, single-cycle training failed to elicit an increased pyruvate accumulation in the MB vertical lobes compared to non-associative training when ALAT was knocked down in adult cortex glia (Fig. 3f). As the cortex glia specifically surround neuronal somata, we next hypothesized that alanine is transferred to this MB neuron compartment. If so, alanine could be transaminated into pyruvate in MB neuron somata. We therefore monitored the pyruvate flux in MB neuron somata (Extended Data Fig. 4e). Sodium azide application triggered a faster pyruvate accumulation in MB neuron somata after single-cycle training compared to non-associative training (Extended Data Fig. 4f). Furthermore, as in the axonal compartment, this effect was abolished by *ALAT* knockdown in adult cortex glia (Extended Data Fig. 4f). To further confirm that alanine, and not pyruvate, was transferred to MB neurons for MTM, we knocked down the main monocarboxylate transporters (MCTs) in cortex glia or MB neurons. None of the MCTs tested was necessary for MTM, suggesting that pyruvate is not transferred from cortex glia to MB neurons for MTM (Extended Data Fig. 5). Together, our behavioral and imaging results suggest that the cortex glia produce alanine to sustain neuronal pyruvate needs during MTM and LTM formation.

**Fig. 4:**
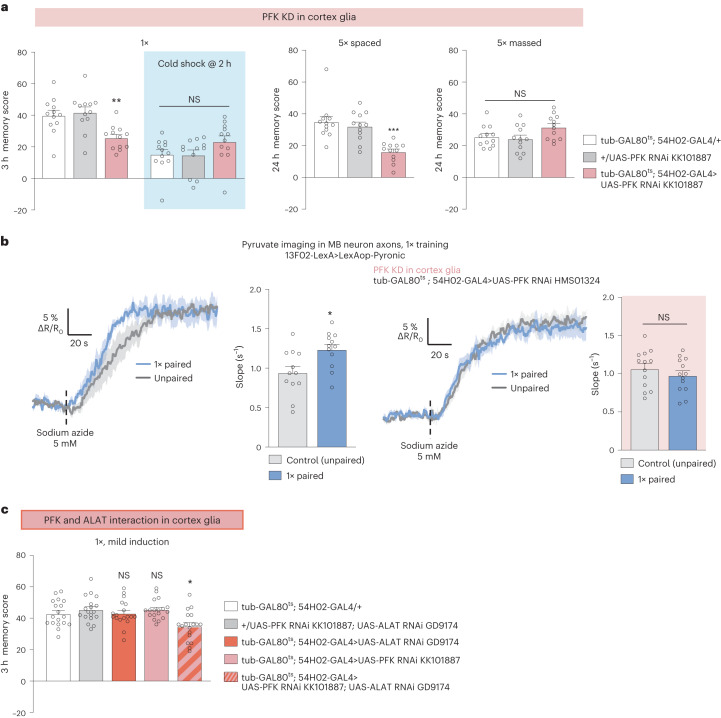
Glycolysis in cortex glia is necessary for MTM and LTM. **a**, *PFK* KD in adult cortex glia impaired memory after single-cycle training (*n* = 12, *F*_2,33_ = 8.26, *P* = 0.001) and spaced training (*n* = 12, *F*_2,33_ = 14.21, *P* = 0.000035), but did not affect memory after single-cycle training followed by cold shock (*n* = 12, *F*_2,33_ = 1.99, *P* = 0.15) or massed training (*n* = 12, *F*_2,33_ = 3.16, *P* = 0.055). **b**, Single-cycle training elicited a faster pyruvate accumulation in MB neuron axons following sodium azide application (5 mM) compared to non-associative unpaired training (*n* = 12, *t*_22_ = 2.75, *P* = 0.012). *PFK* KD in adult cortex glia impaired the single-cycle induced increase in pyruvate accumulation in MB neuron axons following sodium azide application (*n* = 13, *t*_24_ = 0.94, *P* = 0.36). **c**, A mild decrease in both *ALAT* and *PFK* expression in cortex glia impaired memory after single-cycle training, whereas the mild induction of RNAi against *ALAT* or *PFK* alone did not affect memory (*n* = 18, *F*_4,85_ = 5.08, *P* = 0.001). All data are presented as mean ± s.e.m. Asterisks (*P < 0.05; **P < 0.01; NS, not significant, *P* > 0.05.) illustrate the significance level of a two-sided *t*-test or of the least significant pairwise comparison following one-way ANOVA. Source data

**Extended Data Fig. 6:**
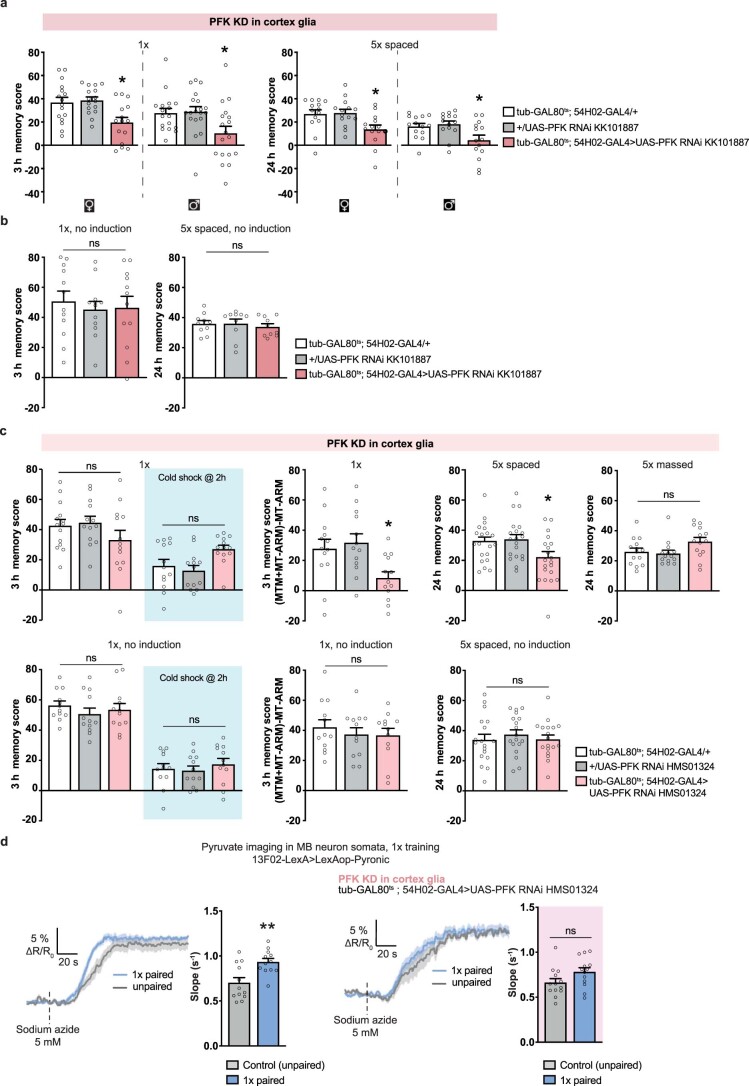
Behavioral controls of *PFK* KD in cortex glia, and pyruvate imaging in *PFK* KD (related to Fig. 4). **a**. *PFK* knockdown (KD) in cortex glia impaired memory in both females and males after single-cycle (females: n = 16, F_2,44_ = 6.89, P = 0.003; males: n = 17 (white bar); n = 19 (gray bar); n = 18 (pink bar), F_2,51_ = 3.22, P = 0.048) and spaced training (females: n = 14, F_2,39_ = 5.04, P = 0.01; males: n = 14, F_2,39_ = 4.95, P = 0.01). **b**. Without induction of *PFK* RNAi, memory after single-cycle (n = 12, F_2,33_ = 0.18, P = 0.84) and spaced training (n = 10, F_2,27_ = 0.24, P = 0.79) were normal. **c**. Behavioral results were confirmed with a second RNAi against PFK. Although no significant effect on memory after single-cycle training was revealed (total: n = 13, F_2,36_ = 1.60, P = 0.22; with cold shock: n = 13, F_2,36_ = 4.56, P = 0.02), subtracting the memory after cold shock from the total memory, which measures only MTM, revealed an MTM impairment (n = 13, F_2,36_ = 5.23, P = 0.01). LTM was also impaired (n = 20, F_2,57_ = 5.10, P = 0.009), but not memory after massed training (n = 14, F_2,39_ = 3.05, P = 0.06). Without induction of *PFK* RNAi, all memories were normal (single-cycle: n = 12, F_2,33_ = 0.60, P = 0.55; single-cycle followed by cold shock: n = 12, F_2,33_ = 0.41, P = 0.67; MTM: n = 12, F_2,33_ = 0.38, P = 0.69; spaced training: n = 18, F_2,51_ = 0.35, P = 0.70). **d**. The increased mitochondrial pyruvate flux elicited by single-cycle training in MB neuron somata (n = 12, t_22_ = 3.36, P = 0.003) was impaired by *PFK* knockdown in adult cortex glia (n = 12, t_22_ = 1.88, P = 0.07). All data are presented as mean ± SEM. Asterisks (*P < 0.05; **P < 0.01; ***P < 0.001; ns: not significant, P > 0.05) illustrate the significance level of two-sided t-test, or of the least significant pairwise comparison following 1-way ANOVA. Source data

Next, we sought to confirm whether alanine production is derived from glucose metabolism in cortex glia. Indeed, knockdown of the gene encoding the glycolytic enzyme PFK in adult cortex glia impaired both MTM and LTM (Fig. 4a and Extended Data Fig. 6a). MTM and LTM were not impaired when *PFK* RNAi was not induced (Extended Data Fig. 6b). LT-ARM was not affected by *PFK* knockdown (Fig. 4a). Sensory acuity for the relevant stimuli was normal in the flies of interest (Supplementary Table 4). These behavioral experiments were replicated using a second, non-overlapping *PFK* RNAi (Extended Data Fig. 6c). In addition, single-cycle training failed to elicit increased pyruvate consumption in the MB vertical lobes or somata compared to non-associative training when *PFK* was knocked down in adult cortex glia (Fig. 4b and Extended Data Fig. 6d). To confirm that glycolysis and alanine transamination in cortex glia act together for MTM formation, we tested whether PFK and ALAT enzyme functionally interact. For this, we optimized the RNAi induction protocol so that simple knockdown of either *PFK* or *ALAT* in cortex glia would not yield any MTM defect, allowing us to test the effect of the double knockdown of *PFK* and *ALAT*. Mild induction of *PFK* and *ALAT* RNAi together in cortex glia impaired MTM, whereas mild induction of *PFK* or *ALAT* RNAi alone had no effect on MTM (Fig. 4c). This result suggests that PFK and ALAT belong to the same pathway allowing MTM formation. These behavioral and imaging results therefore suggest that cortex glia glycolysis is required for alanine production as well as MTM and LTM formation. Collectively, these data demonstrate the existence of a cortex glia-to-MB neuron alanine transfer that is dependent on glial glycolysis to sustain their pyruvate need required by memory formation.

### Two glucose transporters act in cortex glia for memory

**Fig. 5:**
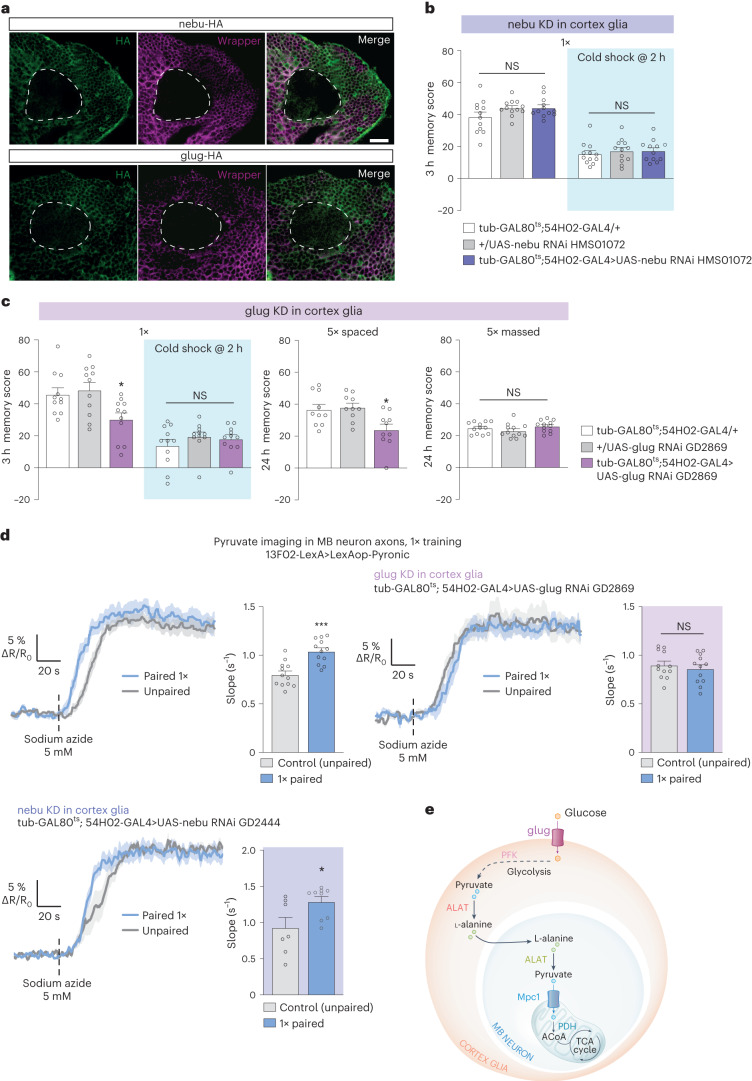
Cortex glia take up hexose through glug specifically for glycolysis-derived alanine synthesis. **a**, Immunohistochemistry of *nebu-HA* (top) and *glug-HA* (bottom) brains showing a *nebu* or *glug* expression pattern (HA, green) within the cortex glia (wrapper, red). The dashed line indicates the MB calyx region. Scale bar, 20 µm. **b**, *Nebu* KD in adult cortex glia did not affect memory after single-cycle training (*n* = 12, *F*_2,33_ = 2.21, *P* = 0.13; after cold shock: *n* = 12, *F*_2,33_ = 0.31, *P* = 0.73) **c**, *Glug* knockdown in adult cortex glia impaired memory after single-cycle training (*n* = 11, *F*_2,30_ = 5.36, *P* = 0.01) and spaced training (*n* = 10, *F*_2,27_ = 5.92, *P* = 0.007), but did not affect memory after single-cycle training followed by cold shock (*n* = 11, *F*_2,30_ = 0.84, *P* = 0.44) or massed training (*n* = 12, *F*_2,33_ = 1.73, *P* = 0.19). **d**, The increase in mitochondrial pyruvate flux elicited by single-cycle training (*n* = 12, *t*_22_ = 4.62, *P* = 0.0001) was impaired by *glug* KD in adult cortex glia (*n* = 12, *t*_22_ = 0.65, *P* = 0.52). but not by *nebu* KD in adult cortex glia (*n* = 7 (control); n = 9 (1×), *t*_14_ = 2.41, *P* = 0.03). **e**, Upon MTM formation, hexose sugar, most likely glucose, taken up by cortex glia through glug is routed to glycolysis, to form pyruvate. Pyruvate is then transaminated to alanine by ALAT. Alanine in MB neurons is transaminated back to pyruvate by ALAT. Pyruvate transported into mitochondria through mitochondrial pyruvate carrier 1 (Mpc1) is transformed into acetyl-CoA (ACoA) by PDH, where it is likely integrated into the TCA cycle to produce energy upon oxidative phosphorylation. All data are presented as mean ± s.e.m. Asterisks (*P < 0.05; ***P < 0.001; NS, not significant, *P* > 0.05) illustrate the significance level of a two-sided *t*-test or of the least significant pairwise comparison following one-way ANOVA. Source data

**Extended Data Fig. 7:**
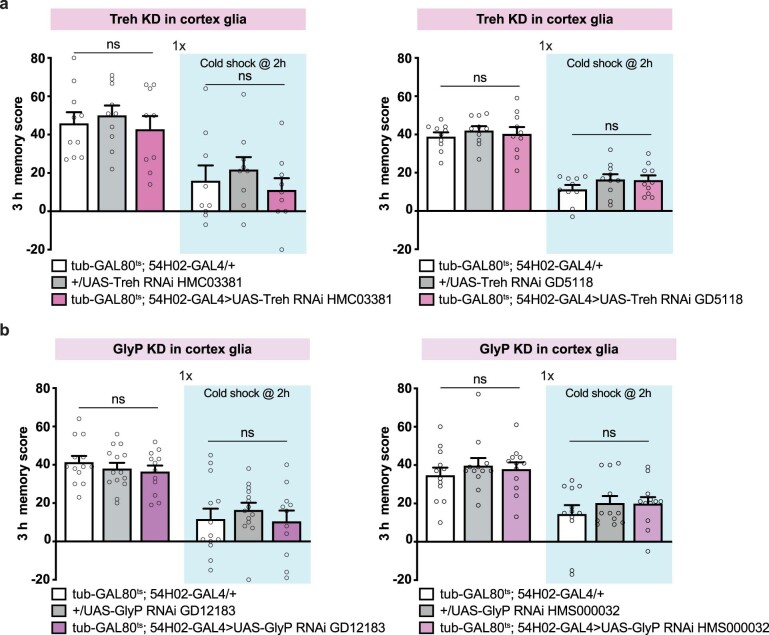
Behavioral controls of *Treh* and *Glyp* KD in cortex glia (related to Fig. 5). **a**. *Treh* knockdown (KD) in adult cortex glia did not affect memory after single-cycle training (RNAi HMC03381: n = 10, F_2,27_ = 0.20, P = 0.82; RNAi GD5118: n = 10, F_2,27_ = 1.15, P = 0.33) or single-cycle training followed by cold shock (RNAi HMC03381: n = 10, F_2,27_ = 0.69, P = 0.51; RNAi GD5118: n = 10, F_2,27_ = 1.32, P = 0.28). **b**. *GlyP* KD in adult cortex glia did not affect memory after single-cycle training (RNAi GD12183: n = 14, F_2,36_ = 0.64, P = 0.53; RNAi HMS000032: n = 12, F_2,33_ = 0.41, P = 0.66) or single-cycle training followed by cold shock (RNAi GD12183: n = 14, F_2,36_ = 0.41, P = 0.67 RNAi HMS000032: n = 12, F_2,33_ = 0.65, P = 0.53). All data are presented as mean ± SEM. Asterisks (**P < 0.01; ***P < 0.001; ns: not significant, p > 0.05) illustrate the significance level of two-sided t-test, or of the least significant pairwise comparison following 1-way ANOVA. Source data

**Extended Data Fig. 8:**
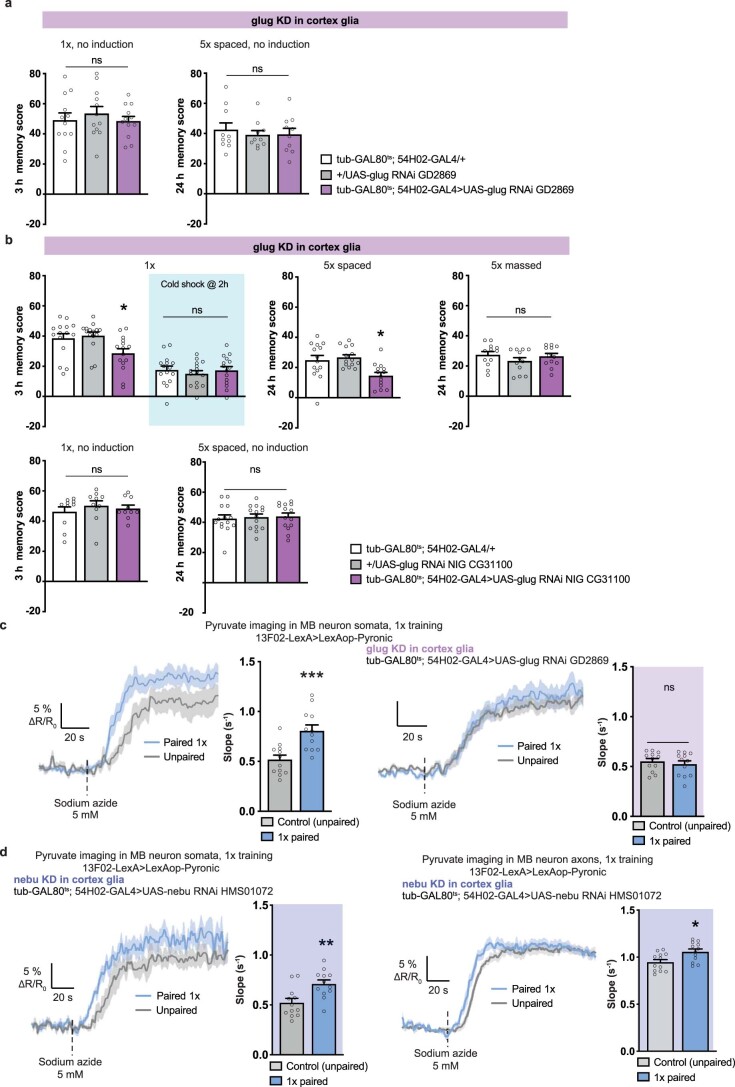
Behavioral controls of *glug* KD in cortex glia and pyruvate imaging in *glug* and *nebu* KD (related to Fig. 5). **a**. Without induction of *glug* RNAi in cortex glia, memory after single-cycle training (n = 12, F_2,33_ = 0.40, P = 0.67) and spaced training (n = 10, F_2,27_ = 0.23, P = 0.79) was normal. **b**. The behavioral experiments were performed with a second RNAi against *glug*. *Glug* knockdown (KD) in adult cortex glia impaired memory after single-cycle training (n = 15, F_2,42_ = 4.88, P = 0.01) and spaced training (n = 14, F_2,39_ = 7.10, P = 0.002), but did not affect memory after single-cycle training followed by cold shock (n = 14, F_2,42_ = 0.32, P = 0.73) or massed training (n = 12, F_2,33_ = 1.06, P = 0.36). Without induction of *glug* RNAi in cortex glia, memory after single-cycle training (n = 10, F_2,27_ = 0.45, P = 0.64) and spaced training (n = 14, F_2,39_ = 1.11, P = 0.90) was normal. **c**. Single-cycle training elicited a faster pyruvate accumulation in MB neuron somata following sodium azide application (5 mM) as compared to non-associative unpaired training (n = 12, t_22_ = 3.89, P = 0.0008). *Glug* KD in adult cortex glia impaired the single-cycle induced increase in pyruvate accumulation in MB neuron somata following sodium azide application (n = 12, t_22_ = 0.66, P = 0.62). **d**. *Nebu* knockdown in adult cortex glia did not impair the single-cycle-induced increase in pyruvate accumulation ta following sodium azide application in MB neuron soma (n = 12, t_22_ = 3.21, P = 0.004) or in MB neuron axons (n = 12, t_22_ = 2.73, P = 0.012). All data are presented as mean ± SEM. Asterisks (**P < 0.01; ***P < 0.001; ns: not significant, P > 0.05) illustrate the significance level of two-sided t-test, or of the least significant pairwise comparison following 1-way ANOVA. Source data

Our results show that glycolysis in cortex glia is required for MTM and LTM. Cortex glia have multiple potential sources of glucose. Indeed, cortex glia can (1) import trehalose as they express the enzyme trehalase^35^, which catalyzes the breakdown of trehalose into glucose; (2) directly take up glucose from the extracellular space^21,36^ to fuel glycolysis; or (3) use glucose-6-phosphate derived from glycogen breakdown^37^. Therefore, we aimed to decipher the origin of glucose in the fueling of glycolysis for MTM formation. Trehalase knockdown in adult cortex glia had no effect on MTM (Extended Data Fig. 7a). Similarly, knocking down glycogen phosphorylase (*GlyP*), the product of which catalyzes the breakdown of glycogen into glucose-1-phosphate, in adult cortex glia had no effect on MTM (Extended Data Fig. 7b). These results suggest that the glucose used for glycolysis-derived alanine production might be directly imported into the cortex glia through a glucose transporter. We previously published that *Drosophila* Glut1, the homolog of mammalian members of the SLC2 membrane transporters Glut1 and Glut3 (ref. ^38^), is responsible for glucose export from cortex glia upon LTM formation, rather than being responsible for glucose import^21^. According to single-cell transcriptomic data, several other members of the SLC2 family of glucose transporters are highly expressed in glial cells^35^. Specifically, among the SLC2 family, two genes encoding predicted hexose sugar transporters are enriched in cortex glia compared to other transporters: *CG31100*, which we named *glug* (for glucose uptake by glia) and *nebu* (https://scope.aertslab.org/#/Davie_et_al_Cell_2018/Davie_et_al_Cell_2018%2FAerts_Fly_AdultBrain_Filtered_57k.loom/gene). To confirm expression of both *glug* and *nebu* in cortex glia, we generated *glug-HA* and *nebu-HA* knock-in lines. Immunohistochemistry experiments on brains of both *glug-HA* and *nebu-HA* flies showed HA patterns matching the honeycomb-like structure of cortex glia, colocalizing with wrapper-positive cortex glia (Fig. 5a). Glug and Nebu therefore represent good candidates for mediating glucose uptake in cortex glia to sustain MTM and LTM. Knockdown of *nebu* in adult cortex glia had no effect on MTM (Fig. 5b). Notably, knockdown of *glug* in adult cortex glia led to MTM and LTM defects, whereas memory after massed training remained intact (Fig. 5c). MTM and LTM were not impaired when *glug* RNAi was not induced (Extended Data Fig. 8a). Sensory acuity for the relevant stimuli was normal in the flies of interest (Supplementary Table 5). These behavioral experiments were replicated using a second *glug* RNAi (Extended Data Fig. 8b).

Next, to test whether *glug* mediates the glucose uptake necessary for glycolysis-derived alanine production aimed at fueling MB neurons, we performed in vivo pyruvate imaging in MB neurons while knocking down *glug* in cortex glia. As seen for *PFK* and *ALAT*, we found that *glug* knockdown in adult cortex glia abolished the increase in pyruvate consumption in MB neuron vertical lobes induced by single-cycle training (Fig. 5d) as well as in MB neuron somata (Extended Data Fig. 8c). Conversely, when *nebu* was knocked down in adult cortex glia, we were still able to observe an increase in pyruvate flux after single-cycle training in MB neuron vertical lobes (Fig. 5d and Extended Data Fig. 8d) and in MB neurons somata (Extended Data Fig. 8d). Together, these data show that the import of glucose into cortex glia via *glug* fuels glial glycolysis to support neuronal pyruvate metabolism (Fig. 5e).

**Fig. 6:**
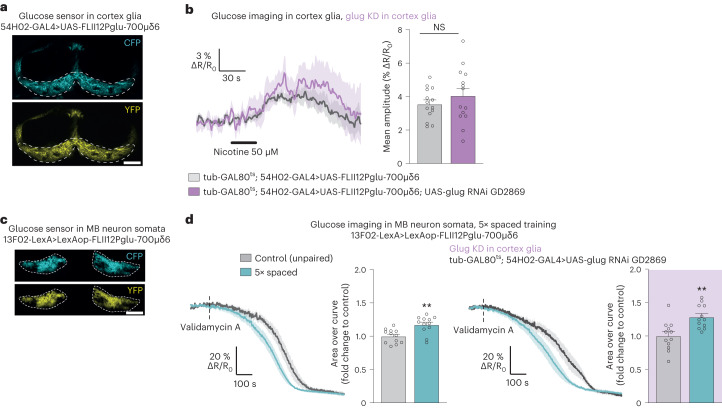
Glug does not mediate glucose transfer to MB neurons for LTM. **a**, The FRET glucose sensor FLII12Pglu-700μδ6^70^ was expressed in adult cortex glia. The two images show the CFP and YFP channels. The dashed lines delimit the cortex glia region where the glucose FRET signal was quantified. Scale bar, 50 µm. **b**, Nicotine stimulation (50 µM, 30 s) increased glucose concentration in cortex glia. KD of *glug* in adult cortex glia had no effect on the nicotine-induced glucose elevation (*n* = 14, *t*_26_ = 0.99, *P* = 0.33). **c**, The FRET glucose sensor FLII12Pglu-700μδ6 was expressed in adult MB neurons. The two images show the CFP and YFP channels. The dashed lines delimit MB neuron somata where the glucose FRET signal was quantified. Scale bar, 30 µm (Supplementary Video 2). **d**, The glucose concentration in MB neuron somata following the application of validamycin A (4 mM, dashed line) decreased faster in flies after spaced training as compared to flies conditioned with a non-associative spaced unpaired training protocol (*n* = 12, *t*_22_ = 3.56, *P* = 0.002). When *glug* was knocked down in adult cortex glia, spaced training continued to elicit a faster decrease of glucose concentration in MB neurons compared to unpaired controls (*n* = 12, *t*_22_ = 3.52, *P* = 0.002). All data are presented as mean ± s.e.m. Asterisks (**P < 0.01; NS, not significant, *P* > 0.05) illustrate the significance level of a two-sided *t*-test. Source data

In addition to providing alanine to fuel neuronal mitochondria for MTM and LTM, cortex glia glucose is involved in a metabolite transfer with neurons that is specific to LTM formation^21^. Indeed, we previously demonstrated that cortex glia activation through the nAchRα7 nicotinic receptor triggers an insulin-dependent increase in glucose concentration, allowing the transfer of glucose to MB neurons to sustain neuronal PPP, specifically for LTM formation^21^. As the glucose transporter mediating glucose import in cortex glia for LTM was not identified at that time, we wondered if the transporter identified here, glug, could also be involved in this process. To test this hypothesis, we performed in vivo glucose imaging experiments in cortex glia in response to nicotine stimulation, using a FRET glucose sensor (Fig. 6a) and our previously well-characterized method^21^. Nicotine application resulted in the expected rise of glucose concentration in cortex glia, whereas *glug* knockdown in adult cortex glia did not affect the nicotine-induced glucose increase (Fig. 6b). To confirm that glug does not mediate acetylcholine-dependent glucose import into cortex glia, we checked that it was not involved in the LTM-specific cortex glia-to-MB neuron glucose shuttle. For this, we performed glucose imaging in MB neuron somata (Fig. 6c) and monitored glucose consumption after spaced training, as previously described^21^. We thus confirmed that spaced training triggers increased glucose consumption by MB neuron somata in normal flies. A similar increase in glucose consumption by MB neuron somata was observed when *glug* was knocked down in cortex glia (Fig. 6d). Together, these data show that glug does not mediate glucose uptake for the LTM-specific cortex glia–MB neuron glucose shuttle.

**Fig. 7:**
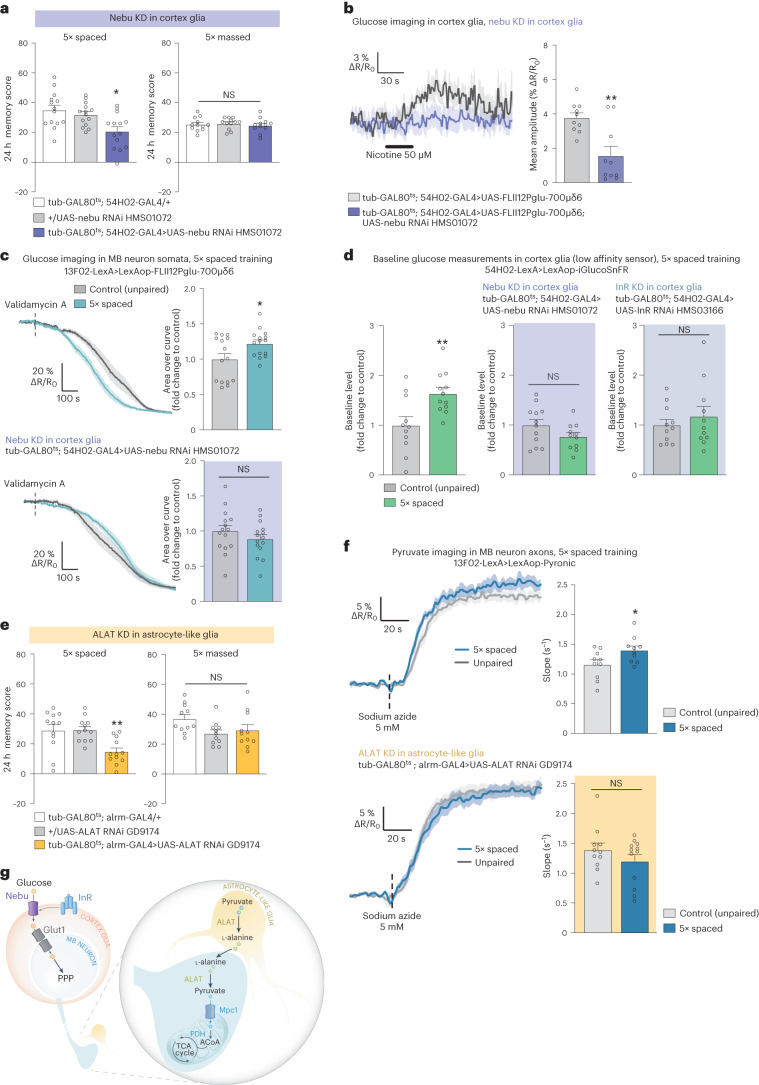
Dual neuron-glia metabolic exchange for LTM. **a**, *Nebu* KD in adult cortex glia impaired memory after spaced training (*n* = 14, *F*_2,39_ = 6.92, *P* = 0.003), but not after massed training (*n* = 12, *F*_2,33_ = 0.25, *P* = 0.78). **b**, Nicotine stimulation (50 µM, 30 s) increased glucose concentration in cortex glia. KD of *nebu* in adult cortex glia impaired the nicotine-induced glucose elevation (*n* = 10, *t*_18_ = 3.70, *P* = 0.002). **c**, The glucose concentration in MB neuron somata following the application of validamycin A (4 mM, dashed line) decreased faster in flies after spaced training compared to flies conditioned with a non-associative spaced unpaired training protocol (*n* = 15, *t*_28_ = 2.38, *P* = 0.025). Spaced training failed to elicit a faster glucose decrease in MB neurons upon *nebu* KD in adult cortex glia (*n* = 15, *t*_28_ = 1.10, *P* = 0.28). **d**, The basal glucose concentration increased in cortex glia following spaced training (*n* = 11–12, *t*_21_ = 3.08, *P* = 0.006). Spaced training failed to increase the basal glucose concentration in cortex glia when either *nebu* or *InR* was knocked down in cortex glia (*nebu* KD, *n* = 12 (control); *n* = 11 (5× spaced), *t*_21_ = 1.64, *P* = 0.12; *InR* KD, *n* = 11, *t*_20_ = 0.77, *P* = 0.45). **e**, *ALAT* KD in adult astrocyte-like glia impaired memory after spaced training (*n* = 12, *F*_2,33_ = 7.69, *P* = 0.002), but did not affect memory after massed training (*n* = 12, *F*_2,33_ = 0.25, *P* = 0.78). **f**, The increased in mitochondrial pyruvate flux elicited by spaced training (*n* = 10, *t*_18_ = 2.32, *P* = 0.03) was impaired by *ALAT* KD in adult astrocyte-like glia (*n* = 11 (control); *n* = 12 (5× spaced), *t*_21_ = 1.18, *P* = 0.25). **g**, Scheme of MB neurons-glia metabolic coupling upon LTM formation. All data are presented as mean ± s.e.m. Asterisks (*P < 0.05; **P < 0.01; NS, not significant, *P* > 0.05) illustrate the significance level of a two-sided *t*-test or of the least significant pairwise comparison following one-way ANOVA. Source data

**Extended Data Fig. 9:**
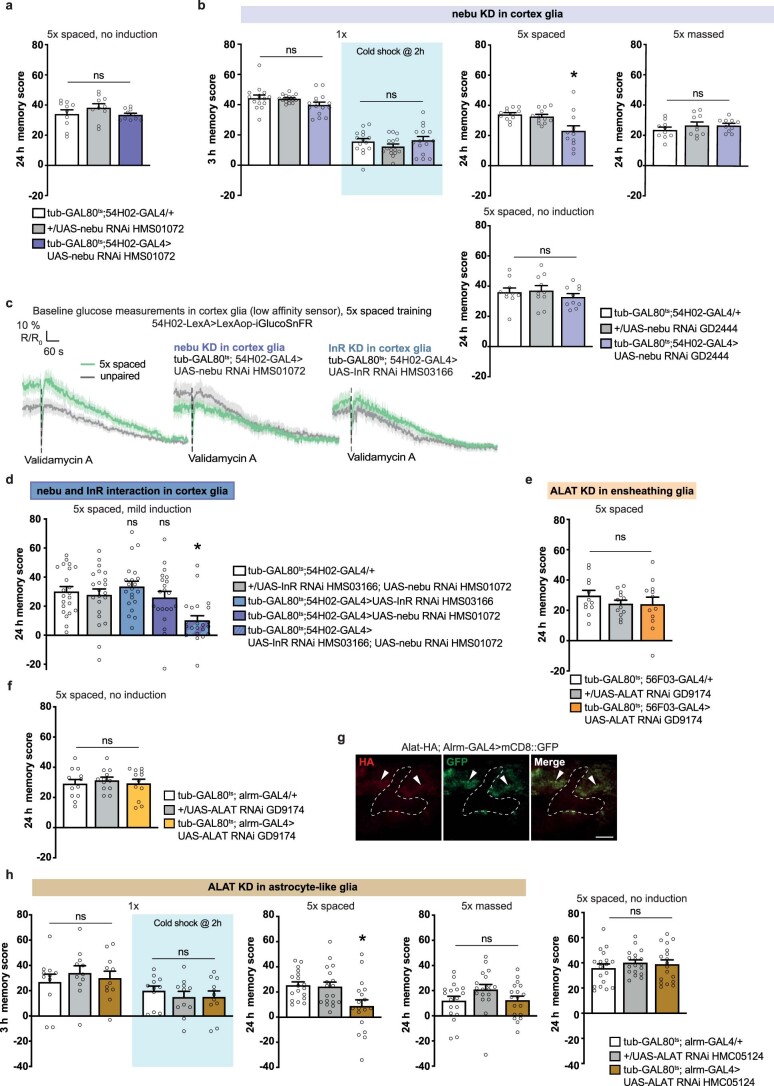
Behavioral controls of *nebu* and *ALTA* KD in glia, and glucose imaging for *nebu* and *InR* (related to Fig. 7). **a**. Without induction of *nebu* RNAi, memory after spaced training (n = 10, F_2,27_ = 1.31, P = 0.29) was normal. **b**. Behavioral results were confirmed with a second RNAi against *Nebu*. LTM was impaired (n = 12, F_2,33_ = 7.47, P = 0.002), but not memory after single-cycle (n = 15, F_2,42_ = 2.40, P = 0.10; cold shock: n = 15, F_2,42_ = 1.15, P = 0.33), or massed training (n = 10, F_2,27_ = 0.96, P = 0.40). Without induction, LTM was normal (n = 10, F_2,27_ = 0.60, P = 0.55). **c**. Glucose concentration measurements corresponding to data in Fig. 7d (see also Supplementary Video 3). Validamycin A (4 mM, dashed line) was applied to measure the sensor floor level, providing an internal reference. **d**. Mild decrease in both *nebu* and *InR* expression in cortex glia impaired memory after spaced training, whereas the separate mild induction of either RNAi did not (n = 22 for all datasets except plain blue and purple bars (n = 21), F_4,103_ = 6.03, P = 0.00021). **e**. *ALAT* KD in ensheathing glia did not affect LTM (n = 12, F_2,33_ = 0.76, P = 0.48). **f**. Without induction of *ALAT* RNAi in astrocyte-like glia, LTM was normal (n = 12, F_2,33_ = 0.23, P = 0.80). **g**. Immunohistochemistry of *ALAT-HA; Alrm-GAL4* > *mCD8::GFP* brain (dashed line delimits MB lobes). Scale bar: 20 µm. **h**. Behavioral experiments with a second RNAi against *ALAT* in astrocyte-like glia. LTM was impaired (n = 18, F_2,51_ = 5.76, P = 0.006), but not memory after single-cycle (n = 11, F_2,30_ = 0.38, P = 0.69; cold shock: n = 11, F_2,30_ = 0.43, P = 0.66), or massed training (n = 18, F_2,51_ = 1.96, P = 0.15). Without RNAi induction, LTM was normal (n = 18, F_2,51_ = 0.53, P = 0.59). All data are presented as mean ± SEM. Asterisks (*P < 0.05; **P < 0.01; ***P < 0.001; ns: not significant, P > 0.05) illustrate the least significant pairwise comparison following 1-way ANOVA. Source data

We therefore hypothesized that another glucose transporter might mediate glucose uptake in cortex glia specifically for LTM. Knockdown of *nebu* in adult cortex glia impaired LTM (Fig. 7a). LTM was not impaired when *nebu* RNAi was not induced (Extended Data Fig. 9a). Sensory acuity for the relevant stimuli was normal in the flies of interest (Supplementary Table 6). These results were confirmed with a second, non-overlapping *nebu* RNAi (Extended Data Fig. 9b). As nebu is specifically required for LTM, we tested whether it mediates the nicotine-induced glucose uptake in cortex glia. Indeed, knocking down *nebu* in adult cortex glia dampened the nicotine-induced glucose increase in cortex glia (Fig. 7b). Furthermore, spaced training failed to induce any glucose consumption increase in MB neurons when *nebu* was knocked down in cortex glia (Fig. 7c). We previously showed that this glucose transfer was linked to a transient increase in glucose concentration in cortex glia upon LTM formation^21^. By expressing a low-affinity glucose sensor in cortex glia^21^, we show here that nebu is necessary for the increase in glucose level in cortex glia upon LTM formation (Fig. 7d and Extended Data Fig. 9c). The cortex glia–MB neuron glucose shuttle depends on insulin receptor activation in cortex glia. Spaced training failed to increase glucose levels in cortex glia when insulin receptor *InR* was knocked down in cortex glia (Fig. 7d and Extended Data Fig. 9c). These data suggest that during LTM formation, nebu acts downstream of InR activation to import glucose into cortex glia, to sustain MB neuron glucose needs for the PPP. To strengthen this hypothesis, we tested the functional interaction of nebu and InR. A mild decrease in *nebu* and *InR* expression together led to an LTM defect, whereas a mild decrease in either nebu or InR expression alone left LTM intact (Extended Data Fig. 9d). Collectively, these results reveal that glial glycolysis for MTM and LTM and LTM-specific glucose import are supported by two different transporters in cortex glia (glug and nebu).

### Astrocyte-derived alanine fuels MB neurons for LTM formation

As LTM relies on highly energy-demanding processes^39^, we wondered whether it could require additional metabolic resources compared to MTM. In mammals, LTM is sustained by energy fuel provided to neurons by astrocytes^8^. We thus asked whether astrocytes in our model participate in LTM formation by sustaining neuronal energy needs. As MB neurons do not need glycolysis or LDH activity for LTM (Fig. 2b,c), we hypothesized that energy might be provided to neurons in the form of alanine for LTM. Indeed, knockdown of *ALAT* in astrocytes, but not in ensheathing glia impaired LTM (Fig. 7e, Extended Data Fig. 9e,f and Supplementary Table 6). We confirmed by immunohistochemistry experiments that *ALAT* was expressed in astrocyte-like glia (Extended Data Fig. 9g). LT-ARM was not impaired by knockdown of *ALAT* in astrocytes (Fig. 7e). These behavioral experiments were replicated using the other *ALAT* RNAi (Extended Data Fig. 9h). To test whether astrocyte-derived alanine is used by MB neurons as an energy substrate for LTM, we performed pyruvate imaging experiments upon spaced training. Pyruvate consumption by MB neurons was increased upon LTM formation, as we previously demonstrated^23^ (Fig. 7f). Notably, spaced training failed to increase pyruvate consumption by MB neurons when *ALAT* was knocked down in astrocyte-like glia (Fig. 7f). Collectively, these data show that astrocyte-like glia-derived alanine sustains neuronal pyruvate needs for LTM formation.

## Discussion

In this study, we investigated how glia-neuron metabolic coupling meets the energy demands of memory formation in *Drosophila*. We found that glucose drawn from the extracellular space by cortex glia is used to produce glycolysis-derived alanine. Alanine is in turn used by MB neurons as an energy substrate, which fuels the increased pyruvate mitochondrial uptake occurring after both single-cycle and spaced training and sustains the formation of MTM and LTM. Combined, these processes constitute a cortex glia–MB neuron alanine transfer at the root of memory formation (Fig. 5e). We revealed that on top of glucose supporting the alanine shuttle, the cortex glia mobilize, via the nebu transporter, an additional flow of glucose specifically for LTM, fueling the PPP in MB neurons^21^. In parallel, astrocyte-like glia provide additional alanine for LTM (Fig. 7g). The model we describe raises the following questions: why is alanine used as an alternative energy substrate for memory instead of lactate, which has been described as necessary for memory formation in rats^8^? And is alanine used as an energy substrate in other contexts?

Several pieces of evidence point to the potential use of alanine as a neuronal energy substrate in other species. In *PDH*-deficient mice, which have disrupted mitochondrial metabolism, NMR spectroscopy analysis has revealed the accumulation of alanine in the brain, suggesting that it is a substrate that normally fuels mitochondrial metabolism^40^. In support of this study, it was confirmed ex vivo that alanine can be produced from glucose in the murine hippocampus^41^, although the cellular origin of alanine was not identified. *ALAT* is expressed by both neurons and astrocytes in the mouse and human brain^42,43^. Measurements of metabolic activity of cultured cortical neurons has revealed substantial alanine uptake from neurons^17^. Moreover, cultured cortical neurons produce CO_2_ from alanine, indicating that they can metabolize it for energy purposes. In parallel, cultured astrocytes release alanine produced from glucose^17,44^. These features are consistent with the existence of an alanine transfer from glial cells to neurons, at least in basal conditions. Our study takes this further by showing that this alanine shuttling is physiologically relevant and specifically activated in vivo upon memory formation. In support of our data, previous NMR studies have demonstrated that glucose-derived alanine is enriched in activated areas of the rat brain upon sensory stimulation^45,46^. An activity-dependent glia-to-neuron alanine shuttle was further described in studies using ex vivo honeybee retina preparations, in which stimulation of photoreceptors triggered an increase in alanine use for energy production^47^. In the same model, the alanine source is the neighboring glial cells^16^. A similar system exists in the mammalian retina in which specialized astrocytes, namely Müller cells, transaminate pyruvate into alanine in response to glutamate stimulation. Collectively, these studies support the existence of a glia–neuron alanine shuttle across several species, similar to the cortex glia–MB neuron shuttle that we have described for memory formation in *Drosophila*. Notably, neither these studies nor our present work have identified the amino acid transporter that mediates the glia-to-neuron alanine transfer. In humans, several *ALAT* mutations have been associated with intellectual disability, revealing the importance of alanine metabolism for cerebral functions^48–50^. Last, increased alanine metabolism has been correlated with the development of the nervous system. Indeed, *ALAT* expression is increased at the peaks of synaptogenesis in the developing mouse hippocampus^50^. Additionally, the *ALAT* knockout mouse model exhibits decreased TCA cycle activity associated with a decreased brain size and reduced synapse formation^50^. This supports the participation of the glia–neuron alanine shuttle in other energy-costly cerebral processes in addition to memory.

By monitoring neuronal pyruvate consumption in vivo, we previously demonstrated that pyruvate metabolism in neurons is increased following LTM formation^23^. This raised the question of the nature of the fuel enabling pyruvate metabolism in MB neurons. In a more recent study, we ruled out neuronal glycolysis as the source of MB neuron pyruvate for LTM^21^. Here, we demonstrated that pyruvate uptake in MB neuron mitochondria was necessary for both LTM and MTM. The upregulation of pyruvate metabolism in MB neurons depends on ALAT activity, suggesting that alanine is the substrate for pyruvate production. Here, glia provide alanine to sustain upregulated pyruvate consumption in MB neurons for LTM. In contrast, studies conducted in rats have shown that LTM depends on lactate import from astrocytes^8^. This brings into question what advantage producing alanine instead of lactate could offer. It should be noted that the ANLS model was initially proposed to account for astrocyte–neuron coupling at glutamatergic synapses. Glycolysis and lactate production in astrocytes are directly coupled, as LDH regenerates NAD^+^ from the NADH produced during glycolysis. In parallel, astrocytes can oxidize glutamate to produce NADH as well as TCA cycle intermediates to sustain their own energy needs^51,52^. Indeed, glutamate is a substantial energy substrate in astrocytes as it contributes to more than 20% of astrocytic oxidative metabolism^53,54^. In non-glutamatergic systems, it is likely that glial cells need alternative sources to sustain their energy metabolism. In the absence of LDH activity, glycolytic NADH can be spared for glial metabolic needs. Furthermore, the transamination reaction of pyruvate into alanine catalyzed by ALAT does not require the conversion of NADH to NAD^+^. Therefore, producing alanine instead of lactate in glial cells would allow saving glycolysis-derived NADH for glial ATP production. This would make the alanine shuttle an efficient mechanism to sustain the energy needs of glial cells and neighboring non-glutamatergic neurons. In support of such a neuron subtype-specific utilization of alanine, studies conducted in vitro show that GABAergic neurons display a higher net alanine uptake rate than glutamatergic neurons^44^. Here, we describe a model in which alanine is the end-product of glial glycolysis, fueling cholinergic MB neurons. Interestingly, nicotinic receptor activation leads to a potassium efflux, which has been shown to activate glial glycolysis^55,56^. This could be a way to enable metabolic transfer in an activity-dependent manner. The alanine shuttle could therefore be a relevant mechanism that sustains the energy needs of cholinergic neurons.

ALAT transamination consists of alanine–pyruvate conversion coupled to α-ketoglutarate–glutamate conversion. We show that glial ALAT catalyzes the reaction in the direction of alanine production, whereas neuronal ALAT catalyzes the reaction in the opposite direction (alanine to pyruvate). These reactions should result in a parallel production of α-ketoglutarate in glia and glutamate in neurons. In both cell types, glutamate dehydrogenase could be involved in the regeneration of glutamate or α-ketoglutarate, respectively. Such reactions would produce ammonium ions in neurons and conversely consume ammonium ions in glia. Neuronal ammonium is released and uptaken by neighboring glial cells in both mammals^57^ and honeybees^58^. Notably, ammonium activates astrocyte metabolism in rodents^59,60^ and has been shown to promote glial alanine production and release in the honeybee retina^58^. Therefore, in our model, the ammonium produced by neurons upon alanine transamination into pyruvate could maintain a positive loop to sustain glia-to-neuron alanine transfer.

We previously revealed the existence of a nicotinic acetylcholine receptor-dependent glucose shuttle from cortex glia to MB neurons that fuels the neuronal PPP, which is crucial for LTM formation^21^. Here we identify nebu, another member of the SLC2 family that is also highly expressed in cortex glia according to single-cell transcriptomic profiling in the fly brain^35^, as necessary for LTM specifically. In addition, nebu was necessary for nicotine-induced glucose uptake in cortex glia, increased glucose consumption in MB neurons and increased glucose concentration in cortex glia following LTM conditioning. Notably, we previously reported that the glucose shuttle from cortex glia to MB neurons for LTM was dependent on insulin signaling^21^. Indeed, the glucose concentration increase in cortex glia depends on insulin peptide Ilp4, which acts autocrinally on its receptor InR in cortex glia. Insulin-dependent glucose uptake has been reported in mammalian peripheral organs and the brain, where it acts through the insulin-sensitive glucose transporter Glut4 (refs. ^61,62^). Similar to what occurs in mammalian adipose tissue in response to insulin, InR signaling in *Drosophila* fat cells allows glucose uptake and storage^63,64^; however, to date, no equivalent glucose transporter responding to insulin has been identified in *Drosophila*^63^. Here, we demonstrate that the glucose transporter nebu and the insulin receptor belong to the same pathway and act together to increase glucose concentration in cortex glia, suggesting that nebu functions as an insulin-sensitive glucose transporter in *Drosophila*.

Overall, this work, along with our recent demonstration that glia provide glucose to neurons for the PPP, provides a global picture of glucose metabolism underlying memory formation in *Drosophila*, highlighting the existence of two parallel fates for glucose orchestrated by cortex glia.

## Methods

### Experimental model

Flies (*D.* *melanogaster*) were raised on standard medium at 18 or 23 °C (depending on the experiments; see respective details below) and 60% humidity in a 12-h light–dark cycle. The study was performed on 1–4-day-old adult flies. For behavior experiments, both mated male and female flies were used. For imaging and biochemistry experiments, mated female flies were used because of their larger size. All flies obtained from libraries or received after the injection of transgenes (except flies from the TRiP RNAi collection) were outcrossed for five generations to a reference strain carrying the w^1118^ mutation in an otherwise Canton Special (Canton S) genetic background. Because TRiP RNAi transgenes are labeled by a y^+^ marker, these lines were outcrossed to a y^1^w^67*c*23^ strain in an otherwise Canton S background. All strains used in this study are described in Supplementary Table 8.

### Behavior experiments

For behavior experiments, flies were raised on standard medium at 18 °C and 60% humidity in a 12-h light–dark cycle. We used the TARGET system^29^ to inducibly express RNAi constructs exclusively in adult flies and not during development. To achieve the induction of RNAi expression, adult flies were kept at 30.5 °C for 3 d before conditioning. To test for genetic interactions, flies were kept at 30.5 °C for 36 h before conditioning. Otherwise, experimental flies (0–3 d old) were transferred to fresh bottles containing standard medium 24 h before conditioning.

The behavior experiments, including the sample sizes, were conducted similarly to other studies from our laboratory^21,23,65^. Groups of 20–50 flies (females and males, mated) were subjected to one of the following olfactory conditioning protocols: a single cycle (1× training), five consecutive associative training cycles (5× massed training), or five associative cycles spaced by 15-min inter-trial intervals (5× spaced training). Non-associative control protocols (unpaired protocols) were also employed for imaging experiments. Conditioning was performed using previously described barrel-type machines that allow the parallel training of up to six groups. Throughout the conditioning protocol, each barrel was plugged into a constant air flow at 2 l min^−1^. For a single cycle of associative training, flies were first exposed to an odorant (the CS^+^) for 1 min while 12 pulses of 5-s long 60 V electric shocks were delivered; flies were then exposed 45 s later to a second odorant without shocks (the CS^−^) for 1 min. The odorants 3-octanol and 4-methylcyclohexanol, diluted in paraffin oil to a final concentration of 2.79 × 10^−1^ g l^−1^, were alternately used as conditioned stimuli. During unpaired conditionings, the odor and shock stimuli were delivered separately in time, with shocks occurring 3 min before the first odorant.

Flies were kept on standard medium between conditioning and the memory test, either at 25 °C for flies tested 3 h after training or at 18 °C for flies tested 24 h after training. To test for anesthesia-resistant memory after 1× training, flies were subjected to cold treatment exposure (4 °C for 2 min) 1 h before testing. The memory test was performed in a T-maze apparatus, typically 3 h after single-cycle training or 24 h after massed or spaced training. Each arm of the T-maze was connected to a bottle containing 3-octanol and 4-methylcyclohexanol, diluted in paraffin oil to a final concentration identical to the one used for conditioning. Flies were given 1 min to choose between either arm of the T-maze. A performance score was calculated as the number of flies avoiding the conditioned odor minus the number of flies preferring the conditioned odor, divided by the total number of flies. A single performance index value is the average of two scores obtained from two groups of genotypically identical flies conditioned in two reciprocal experiments, using either odorant (3-octanol or 4-methylcyclohexanol) as the CS^+^. The indicated ‘*n*’ is the number of independent performance index values for each genotype.

The shock response tests were performed at 25 °C by placing flies in two connected compartments; electric shocks were provided in only one of the compartments. Flies were given 1 min to move freely in these compartments, after which they were trapped, collected and counted. The compartment where the electric shocks were delivered was alternated between two consecutive groups. Shock avoidance was calculated as for the memory test.

Because the delivery of electric shocks can modify olfactory acuity, our olfactory avoidance tests were performed on flies that had first been presented another odor paired with electric shocks. Innate odor avoidance was measured in a T-maze similar to those used for memory tests, in which one arm of the T-maze was connected to a bottle with odor diluted in paraffin oil and the other arm was connected to a bottle with paraffin oil only. Naive flies were given the choice between the two arms for 1 min. The odor-interlaced side was alternated for successively tested groups. Odor concentrations used in this assay were the same as for the memory assays. At these concentrations, both odorants are innately repulsive.

### Alanine feeding

Flies were transferred on regular medium supplemented with l-alanine (Sigma-Aldrich, cat. no. 05129) to a final concentration of 60 mM for 72 h at 30.5 °C (to achieve RNAi induction). The behavior experiment was performed as described in the Methods.

### In vivo pyruvate imaging

Pyruvate imaging experiments were performed on flies expressing the pyruvate sensor in MB neurons via the VT30559-GAL4 driver or 13F02-LexA driver, in combination with either UAS-Pyronic^23^ or LexAop-Pyronic (this study). RNAis were expressed in MB neurons using the inducible tub-GAL80^ts^; VT30559-GAL4 driver or in cortex glia using the inducible tub-GAL80^ts^; 54H02-GAL4 driver. For imaging experiments, flies were raised at 23 °C to increase the expression level of genetically encoded sensors. To achieve the induction of RNAi expression, adult flies were kept at 30.5 °C for 3 d before conditioning.

As in all previous imaging work from our laboratory, all in vivo imaging was performed on female flies, which are preferred because their larger size facilitates surgery. Data were collected indiscriminately from 30 min to 1.5 h after 1× training. A single fly was picked and prepared for imaging as previously described^66^. Briefly, the head capsule was opened and the brain was exposed by gently removing the superior tracheae. The head capsule was bathed in artificial hemolymph solution for the duration of the preparation. The composition of this solution was NaCl 130 mM (Sigma cat. no. S9625), KCl 5 mM (Sigma cat. no. P3911), MgCl_2_ 2 mM (Sigma cat. no. M9272), CaCl_2_ 2 mM (Sigma cat. no. C3881), d-trehalose 5 mM (Sigma T cat. no. 9531), sucrose 30 mM (Sigma cat. no. S9378) and HEPES hemisodium salt 5 mM (Sigma cat. no. H7637). At the end of surgery, any remaining solution was absorbed and a fresh 90-μl droplet of this solution was applied on top of the brain. Two-photon imaging was performed using a Leica TCS-SP5 upright microscope equipped with a ×25, 0.95 NA water-immersion objective. Two-photon excitation was achieved using a Mai Tai DeepSee laser tuned to 825 nm. The frame rate was one or two images per second. Image acquisition was performed using the LAS AF software (Leica Microsystems). Measurements of pyruvate consumption were performed according to a previously well-characterized protocol^23^. After 1 min of baseline acquisition, 10 µl of a 50 mM sodium azide solution (Sigma cat. no. 71289; prepared in the same artificial hemolymph solution) was injected into the 90-µl droplet bathing the fly’s brain, bringing sodium azide to a final concentration of 5 mM.

For the analysis of all pyruvate imaging experiments, regions of interest (ROIs) were delimited by hand around each visible MB vertical lobes or MB neuron somata, and the average intensity of the mTFP and Venus channels over each ROI was calculated over time after background subtraction. The Pyronic sensor was designed so that FRET from mTFP to Venus decreases when pyruvate concentration increases. To obtain a signal that positively correlates with pyruvate concentration, the inverse FRET ratio was computed as mTFP intensity divided by Venus intensity. This ratio was normalized by a baseline value calculated over the 30 s preceding drug injection. The slope was calculated between 10 and 70% of the plateau, while the rise time was calculated from *t*_0_ to the time reaching 70% of the plateau. The indicated ‘*n*’ is the number of animals that were assayed in each condition.

### In vivo glucose imaging

Glucose imaging experiments were performed on flies expressing the glucose sensor in cortex glia using the 54H02-GAL4 driver or in MB neurons via the 13F02-LexA driver, in combination with either UAS-FLII12Pglu-700μδ6 (ref. ^23^) or LexAop-FLII12Pglu-700μδ6 (ref. ^23^). RNAis were expressed in cortex glia using the inducible tub-GAL80^ts^; 54H02-GAL4 driver. Fly preparation was performed as described for in vivo pyruvate imaging. Two-photon excitation was achieved using a Mai Tai DeepSee laser tuned to 820 nm. The frame rate was one image per second.

Nicotine stimulation experiments were performed as previously described^21^. On each experimental day, nicotine was freshly diluted from a commercial liquid (Sigma N3876) into the saline used for imaging. A perfusion setup at a flux of 2.5 ml min^−1^ enabled the time-restricted application of 50 μM nicotine on top of the brain. A baseline recording was performed for 1 min, after which the saline supply was switched to drug supply. The solution reached the in vivo preparation within 30 s. The stimulation was maintained for 30 s before switching back to the saline perfusion for an additional 5 min.

Glucose consumption experiments were performed as previously described^21^. Validamycin A (Sigma 32347), a trehalase-selective inhibitor, was directly diluted into artificial hemolymph solution at a final concentration of 40 mM, aliquoted, and stored at −20 °C. A freshly thawed aliquot was used for every fly. After 1 min of baseline acquisition, 10 μl of the solution was added to the 90-μl saline droplet on top of the brain, bringing validamycin A to a final concentration of 4 mM. The signal was then acquired for another 12 min. Data were collected indiscriminately from 30 min to 2 h after 5× spaced training. Image analysis was performed as previously described^21^. ROIs were delimited by hand around the labeled regions of interest (cortex glia or MB neuron somata). The average intensity of the YFP and CFP channels over each ROI was calculated over time after background subtraction. The FRET ratio (YFP/CFP) of the FLII12Pglu-700μδ6 glucose sensor was computed to obtain a signal positively correlated with the glucose concentration. This ratio was normalized by a baseline value calculated over the 30 s preceding drug injection.

For the glucose consumption experiments, the area over the curve (AOC) was computed to obtain values positively correlated with glucose consumption. The AOC was calculated as the integral between 200 s and 900 s.

To measure basal glucose levels in cortex glia, glucose imaging experiments were performed in cortex glia by expressing the low-affinity glucose sensor iGlucoSnFR (LexAop-iGlucoSnFR^67^) via the 54H02-LexA driver as previously described^21^. Fly preparation was performed as described for in vivo imaging. Two-photon excitation was achieved using a Mai Tai DeepSee laser tuned to 905 nm. The frame rate was one image per second. To estimate the baseline glucose concentration in cortex glia, the trace of fluorescence decrease following validamycin A application was normalized to the value of the stable floor plateau that ended all recordings, corresponding to the fully unbound state of the sensor. The decrease was recorded for 900 s after the drug application, and the floor value was set as the average over the last 150 s of the recording. The baseline glucose level was measured during the 90 s preceding validamycin A application. The indicated ‘*n*’ is the number of animals that were assayed in each condition.

### ALAT activity measurements

ALAT activity was measured using a commercial colorimetric assay (ALT assay kit, Sigma-Aldrich, cat. no. MAK052), according to the manufacturer’s instructions. For each genotype, 100 females were snap frozen in liquid nitrogen and heads were separated from bodies. Heads were then homogenized in the manufacturer’s buffer, and the resulting samples were diluted 1:4 for the assay. Samples were read in a FlexA-200 microplate spectrophotometer (Allsheng) at 570 nm, alongside a pyruvate standard and the positive control, according to the manufacturer’s instructions. To assess ALAT-specific activity, measured ALAT activity was normalized to the measured protein content by performing a Lowry assay (Thermo Fisher Scientific, cat. no. 23240), according to the manufacturer’s instructions.

### Immunohistochemistry

Before dissection, 2–4-day-old female flies were fixed in 4% paraformaldehyde in PBST (PBS containing 1% Triton X-100) at 4 °C overnight. Fly brains were dissected on ice in PBS solution, fixed for 1 h in 4% paraformaldehyde PBST, and rinsed three times for 20 min in PBST. Brains were blocked with 2% BSA in PBST for 2 h before incubation with primary antibodies in the blocking solution (2% BSA in PBST) at 4 °C overnight. The following primary antibodies were used: rat anti-HA (1:400 dilution; Roche, cat. no. 11867423001), mouse anti-nc82 (1:100 dilution, DSHB), mouse anti-wrapper (1:100 dilution, DSHB) and rabbit anti-GFP (1:400 dilution; Thermo Fisher Scientific, A11122). The following day, brains were rinsed three times for 20 min with PBST and then incubated for 3 h at room temperature with the respective secondary antibodies diluted in blocking solution. The following secondary antibodies were used: anti-rat conjugated to Alexa Fluor 488 (1:400 dilution; Invitrogen, A11006), anti-mouse conjugated to Alexa Fluor 594 (1:400 dilution; Invitrogen, A11005), anti-rat conjugated to Alexa Fluor 594 (1:400 dilution; Invitrogen, A11007) and anti-rabbit conjugated to Alexa Fluor 488 (1:400 dilution; Invitrogen, A11034). Brains were then rinsed once in PBST for 20 min and twice in PBS for 20 min. After rinsing, brains were mounted using Prolong Mounting Medium (Invitrogen). Acquisitions were made using a Nikon A1R confocal microscope, with a ×60/1.40 oil immersion objective.

### Quantitative PCR

The efficiency of the knockdowns used in this study was validated by quantitative PCR with reverse transcription (RT–qPCR) to measure the messenger RNA of the target gene. Female flies carrying the elav-Gal4 pan-neuronal driver or the repo-Gal4 pan-glial driver were crossed either with males carrying the specified UAS-RNAi or with CS males. Fly progeny were reared at 25 °C throughout their development. Then, 0–1-day-old flies were transferred to fresh food for 1 d before RNA extraction. RNA extraction and complementary DNA synthesis were performed as described elsewhere^68,69^ using the same reagents: the RNeasy Plant Mini kit (QIAGEN), RNA MinElute Cleanup kit (QIAGEN), oligo(dT)20 primers and the SuperScript III First-Strand kit (Life Technologies). The level of cDNA for each gene of interest was compared against the level of the α-Tub84B (Tub, CG1913) reference cDNA. Amplification was performed using a LightCycler 480 (Roche) and the SYBR Green I Master mix (Roche). Reactions were carried out in triplicate. The specificity and size of amplification products were assessed by melting curve analyses. Expression relative to the reference was expressed as a ratio (2^−ΔCp^, where Cp is the crossing point). The primers used in this study are described in Supplementary Table 9.

Results of RT–qPCR data are presented in Supplementary Table 7. For KDs that did not yield memory impairments (negative results), both RNAis were checked by qPCR. For KDs that induced memory impairments with two distinct RNAis, at least one of the two RNAi lines was validated. Exceptions were for those RNAi constructs that were already validated by RT–qPCR in previous studies, in which case the corresponding reference is indicated, as well as the reported decrease in mRNA levels.

### Generation of transgenic flies

The 2545_pcDNA3.1(-)Pyronic plasmid^30^ was digested by BamHI and BclI. The resulting 2,287-bp fragment was purified by electrophoresis and cloned into a pJFRC19 plasmid (13XLexAop2-IVS-myr::GFP) previously digested by BglII and XbaI to remove the myr::GFP sequence. The resulting construct was verified by restriction. Transgenic fly strains were obtained by site-specific embryonic injection of the resulting vector in the attP18 landing site (chromosome 1), which was outsourced to Rainbow Transgenic Flies.

HA-tag knock-in lines were generated using the CRISPR technique, outsourced to Rainbow Transgenic Flies. ALAT-HA was generated by inserting the 3×HA sequence

(TACCCATACGATGTTCCTGACTATGCGGGCTATCCCTATGACGTCCCGGACTATGCAGGATCCTATCCATATGACGTTCCAGATTACGCT) and a GS linker after the sixth exon of the ALAT gene, using guide RNA (sequence: CTTGTACTTCTTCATGAACTCGG). Nebu-HA and glug-HA were generated by inserting a 3×HA-3×FLAG sequence (TACCCATACGATGTTCCTGACTATGCGGGCTATCCCTATGACGTCCCGGACTATGCAGGATCCTATCCATATGACGTTCCAGATTACGCTGACTACAAAGACCATGACGGTGATTATAAAGATCATGACATCGATTACAAGGATGACGATGACAAG) followed by a GS linker after the first exon of the nebu gene using gRNA (sequence: GATGCGGTGGACATATCGAAGGG) or the glug gene using gRNA (sequence: ACCACAAGGATTTACCAAAATGG).

### Statistics and reproducibility

All data are presented as mean ± s.e.m. For behavior experiments, two groups of approximately 30 flies were reciprocally conditioned using either octanol or methylcyclohexanol as the CS^+^. The memory score was calculated from the performance of two groups as described above, which represents an experimental replicate. For imaging experiments, one replicate corresponds to one fly brain. For immunostaining (Figs. 2f, 3e and 5a and Extended Data Fig. 9g), representative images are presented but five brains of each conditions were acquired independently. Comparisons of the data series between two conditions were achieved by a two-tailed unpaired *t*-test. Comparisons between more than two distinct groups were made using a one-way ANOVA test, followed by Tukey’s method for pairwise comparisons between the experimental groups and their controls. For alanine feeding rescue experiments, we performed a two-way ANOVA test, followed by Tukey’s post hoc test. ANOVA results are presented as the value of the Fisher distribution *F*(*x*,*y*) obtained from the data, where *x* is the number of degrees of freedom between groups and *y* is the total number of degrees of freedom for the distribution. Statistical tests were performed using the GraphPad Prism v.8 software. In the figures, asterisks illustrate either the significance level of the *t*-test or of the least significant pairwise comparison following an ANOVA, with the following nomenclature: *P < 0.05; **P < 0.01; ***P < 0.001; NS, not significant, *P* > 0.05.

### Reporting summary

Further information on research design is available in the Nature Portfolio Reporting Summary linked to this article.

### Supplementary information

Supplementary Information Supplementary Tables 1–9 and Supplementary Videos 1–3.

Reporting Summary

Supplementary Video 1 Time course of FRET pyruvate imaging in MB neuron axons (VT30559-GAL4>UAS-Pyronic) upon sodium azide (NaN_3_) application (60 s). Top channel, mTFP; bottom channel, Venus.

Supplementary Video 2 Time course of FRET glucose imaging in MB neuron cell bodies (13F02-LexA>LexAop- FLII12Pglu-700μδ6) upon validamycin A (ValA) application (100 s). Top channel, YFP; bottom channel, CFP.

Supplementary Video 3 Time course of glucose imaging in cortex glia (13F02-LexA>LexAop-iGlucoSnFR) upon validamycin A (ValA) application (100 s).

### Source data

Source Data Fig. 1 Statistical Source Data for Fig. 1.

Source Data Fig. 2 Statistical Source Data for Fig. 2.

Source Data Fig. 3 Statistical Source Data for Fig. 3.

Source Data Fig. 4 Statistical Source Data for Fig. 4.

Source Data Fig. 5 Statistical Source Data for Fig. 5.

Source Data Fig. 6 Statistical Source Data for Fig. 6.

Source Data Fig. 7 Statistical Source Data for Fig. 7.

Source Data Extended Data Fig. 1 Statistical Source Data for Extended Data Fig. 1.

Source Data Extended Data Fig. 2 Statistical Source Data for Extended Data Fig. 2.

Source Data Extended Data Fig. 3 Statistical Source Data for Extended Data Fig. 3.

Source Data Extended Data Fig. 4 Statistical Source Data for Extended Data Fig. 4.

Source Data Extended Data Fig. 5 Statistical Source Data for Extended Data Fig. 5.

Source Data Extended Data Fig. 6 Statistical Source Data for Extended Data Fig. 6.

Source Data Extended Data Fig. 7 Statistical Source Data for Extended Data Fig. 7.

Source Data Extended Data Fig. 8 Statistical Source Data for Extended Data Fig. 8.

Source Data Extended Data Fig. 9 Statistical Source Data for Extended Data Fig. 9.

## Data Availability

No datasets that require mandatory deposition into a public database were generated during the current study. Source data that are reported as graphs on figures and extended data figures are available as Supplementary Information alongside the paper. Additional raw data will be shared with no restriction by the corresponding authors upon request. This study made use of the FlyBase database to identify a putative *ALAT* fly ortholog (http://flybase.org/reports/FBgn0030478) and of the SCOPE database of single-cell transcriptomics in the fly brain to identify hexose transporters expressed in cortex glia (https://scope.aertslab.org/#/Davie_et_al_Cell_2018/Davie_et_al_Cell_2018%2FAerts_Fly_AdultBrain_Filtered_57k.loom/gene). Source data are provided with this paper.

## Code availability

This paper does not report original code. Any additional information required to reanalyze the data reported in this paper is available from the corresponding authors upon request.
