# Supplementary material for: Glycolysis-derived alanine from glia fuels neuronal mitochondria for memory in Drosophila
Source: Nat Metab. 2023 Nov 6;5(11):2002–19. doi: 10.1038/s42255-023-00910-y (PMC10663161; doi:10.1038/s42255-023-00910-y)
Supplement: Supplementary file 1 — Supplementary Tables 1–9 and Supplementary Videos 1–3. [file 42255_2023_910_MOESM1_ESM.pdf]

nature metabolism

Article https://doi.org/10.1038/s42255-023-00910-y

Glycolysis-derived alanine from glia fuels neuronal mitochondria for memory in *Drosophila*

In the format provided by the authors and unedited

Supplementary Tables

Supplementary Table 1. Sensory acuity controls related to Figure 1 and Extended Data Figure 1.

Genotypes	Shock reactivity		Olfactory acuity			
			Octanol		Methylcyclohexanol	
	Mean ± SEM	Statistics	Mean ± SEM	Statistics	Mean ± SEM	Statistics
tub-GAL80^{ts}; VT30559-GAL4/+	54.5 ± 4.8	n = 12<br>$F_{2,33}$ = 0.31<br>P = 0.74	57.1 ± 4.9	n = 12<br>$F_{2,33}$= 1.02<br>P = 0.37	52.8 ± 4.9	n = 12<br>$F_{2,33}$ = 0.76<br>P = 0.48
+/UAS-Mpc1 RNAi HMS05634	49.5 ± 3.8		47.9 ± 4.7		49.6 ± 3.1	
tub-GAL80^{ts}; VT30559-GAL4> UAS-Mpc1 RNAi HMS05634	53.0 ± 5.2		55.8 ± 5.2		52.3 ± 4.1	
tub-GAL80^{ts}; VT30559-GAL4/+	68.1 ± 5.2	n = 12<br>$F_{2,33}$ = 1.61<br>P = 0.22	50.6 ± 3.2	n = 12<br>$F_{2,33}$ = 2.70<br>P = 0.08	46.0 ± 5.7	n = 12<br>$F_{2,33}$ = 0.03<br>P = 0.97
+/UAS-PDHE1β RNAi HMC03762	59.8 ± 4.9		50.0 ± 6.3		44.6 ± 5.3	
tub-GAL80^{ts}; VT30559-GAL4> UAS-PDHE1β RNAi HMC03762	71.6 ± 4.2		63.8 ± 4.2		46.5 ± 6.0	
tub-GAL80^{ts}; VT30559-GAL4/+	55.2 ± 3.7	n = 12<br>$F_{2,33}$ = 0.01<br>P > 0.99	56.2 ± 3.5	n = 10<br>$F_{2,27}$ = 1.74<br>P = 0.19	55.2 ± 5.4	n = 10<br>$F_{2,27}$ = 2.22<br>P = 0.14
+/UAS-Mpc1 RNAi KK102734	54.6 ± 5.3		48.2 ± 4.4		42.9 ± 6.6	
tub-GAL80^{ts}; VT30559-GAL4> UAS-Mpc1 RNAi KK102734	54.6 ± 6.5		56.2 ± 2.2		58.7 ± 5.0	

Supplementary Table 2. Sensory acuity controls related to Figure 2 and Extended Data Figure 3.

Genotypes	Shock reactivity		Olfactory acuity			
			Octanol		Methylcyclohexanol	
	Mean ± SEM	Statistics	Mean ± SEM	Statistics	Mean ± SEM	Statistics
tub-GAL80^{ts}; VT30559-GAL4/+	53.4 ± 3.6	n = 10<br>$F_{2,27}$ = 0.25<br>P = 0.78	60.2 ± 5.2	n = 10<br>$F_{2,27}$ = 1.70<br>P = 0.20	70.1 ± 5.0	n = 10<br>$F_{2,27}$ = 0.03<br>P = 0.97
+/UAS-ALAT RNAi GD9174	57.5 ± 5.6		62.7 ± 3.9		71.3 ± 5.8	
tub-GAL80^{ts}; VT30559-GAL4> UAS-ALAT RNAi GD9174	57.9 ± 5.6		71.9 ± 5.0		71.7 ± 4.6	
tub-GAL80^{ts}; VT30559-GAL4/+	54.8 ± 5.1	n = 10<br>$F_{2,27}$ = 0.34<br>P = 0.72	72.3 ± 4.5	n = 10<br>$F_{2,27}$ = 0.48<br>P = 0.70	82.5 ± 3.8	n = 10<br>$F_{2,27}$ = 1.33<br>P = 0.28
+/UAS-ALAT RNAi HMC05124	50.1 ± 3.6		73.7 ± 5.6		75.9 ± 4.6	
tub-GAL80^{ts}; VT30559-GAL4> UAS-ALAT RNAi HMC05124	52.5 ± 3.2		65.7 ± 5.1		80.7 ± 5.7	

Supplementary Table 3. Sensory acuity controls related to Figure 3 and Extended Data Figure 4.

Genotypes	Shock reactivity		Olfactory acuity			
			Octanol		Methylcyclohexanol	
	Mean ± SEM	Statistics	Mean ± SEM	Statistics	Mean ± SEM	Statistics
tub-GAL80ts; 54H02-GAL4/+	62.6 ± 7.0	n = 12<br>$F_{2,33}$= 0.34<br>P = 0.72	68.1 ± 5.1	n = 12<br>$F_{2,33}$ = 0.40<br>P = 0.67	64.9 ± 2.6	n = 12<br>$F_{2,33}$= 1.27<br>P = 0.29
+/UAS-ALAT RNAi GD9174	55.3 ± 6.9		70.2 ± 7.1		72.1 ± 5.3	
tub-GAL80ts; 54H02-GAL4> UAS-ALAT RNAi GD9174	60.9 ± 5.7		75.4 ± 5.3		62.3 ± 5.1	
tub-GAL80ts; 54H02-GAL4/+	62.5 ± 5.0	n = 10<br>$F_{2,27}$ = 0.97<br>P = 0.39	79.4 ± 3.1	n = 10<br>$F_{2,27}$ = 1.40<br>P = 0.26	78.7 ± 3.3	n = 10<br>$F_{2,27}$ = 1.04<br>P = 0.39
+/UAS-ALAT RNAi HMC05124	55.5 ± 5.9		66.6 ± 4.6		78.5 ± 4.8	
tub-GAL80ts; 54H02-GAL4> UAS-ALAT RNAi HMC05124	65.4 ± 4.6		73.3 ± 5.4		80.0 ± 4.6	

Supplementary Table 4. Sensory acuity controls related to Figure 4 and Extended Data Figure 6.

**Genotypes**	**Shock reactivity**		**Olfactory acuity**			
			**Octanol**		**Methylcyclohexanol**	
	**Mean ± SEM**	**Statistics**	**Mean ± SEM**	**Statistics**	**Mean ± SEM**	**Statistics**
tub-GAL80^{ts}; 54H02-GAL4/+	51.7 ± 3.3	n = 10<br>$F_{2,27}$ = 1.24<br>P = 0.30	60.4 ± 4.7	n = 10<br>$F_{2,27}$ =1.95<br>P = 0.14	65.5 ± 4.6	n = 10<br>$F_{2,27}$ = 0.63<br>P = 0.60
+/UAS-PFK RNAi KK101887	59.0 ± 6.0		73.6 ± 4.7		60.1 ± 5.7	
tub-GAL80^{ts}; 54H02-GAL4> UAS-PFK RNAi KK101887	63.1 ± 5.8		60.2 ± 5.1		66.8 ± 5.7	
tub-GAL80^{ts}; 54H02-GAL4/+	66.7 ± 3.4	n = 10<br>$F_{2,27}$ = 0.69<br>P = 0.51	72.4 ± 3.7	n = 10<br>$F_{2,27}$ = 0.58<br>P = 0.63	72.7 ± 5.6	n = 10<br>$F_{2,27}$ = 0.79<br>P = 0.51
+/UAS-PFK RNAi HMS01324	64.8 ± 3.8		74.2 ± 4.4		66.7 ± 4.1	
tub-GAL80^{ts}; 54H02-GAL4> UAS-PFK RNAi HMS01324	60.8 ± 3.6		66.1 ± 5.1		77.1 ± 4.9	

Supplementary Table 5. Sensory acuity controls related to Figure 5 and Extended Data Figure 8.

Genotypes	Shock reactivity		Olfactory acuity			
			Octanol		Methylcyclohexanol	
	Mean ± SEM	Statistics	Mean ± SEM	Statistics	Mean ± SEM	Statistics
tub-GAL80^{ts}; 54H02-GAL4/+	58.9 ± 5.2	n = 10<br>$F_{2,27}$ = 0.55<br>P = 0.65	66.1 ± 5.1	n = 10<br>$F_{2,27}$ = 0.73<br>P = 0.54	62.9 ± 5.4	n = 10<br>$F_{2,27}$ = 0.1<br>P = 0.91
+/UAS-glug RNAi GD2869	65.9 ± 5.5		64.3 ± 4.7		65.9 ± 4.4	
tub-GAL80^{ts}; 54H02-GAL4> UAS-glug RNAi GD2869	61.1 ± 5.9		60.2 ± 4.4		65.5 ± 5.8	
tub-GAL80^{ts}; 54H02-GAL4/+	52.4 ± 2.7	n = 10<br>$F_{2,27}$ = 0.89<br>P = 0.42	44.6 ± 3.8	n = 8<br>$F_{2,21}$ = 0.07<br>P = 0.94	46.3 ± 1.9	n = 8<br>$F_{2,21}$ = 1.76<br>P = 0.20
+/UAS-glug RNAi NIG CG31100	47.5 ± 2.4		44.6 ± 2.9		43.9 ± 2.2	
tub-GAL80^{ts}; 54H02-GAL4> UAS-glug RNAi NIG CG31100	50.7 ± 2.8		43.1 ± 3.3		51.0 ± 3.7	

Supplementary Table 6. Sensory acuity controls related to Figure 7 and Extended Data Figure 9.

Genotypes	Shock reactivity		Olfactory acuity			
			Octanol		Methylcyclohexanol	
	Mean ± SEM	Statistics	Mean ± SEM	Statistics	Mean ± SEM	Statistics
tub-GAL80^{ts}; 54H02-GAL4/+	50.9 ± 3.0	n = 10<br>$F_{2,27}$ = 1.34<br>P = 0.28	73.1 ± 3.7	n = 10<br>$F_{2,27}$ = 0.32<br>P = 0.73	77.6 ± 3.9	n = 10<br>$F_{2,27}$ = 0.25<br>P = 0.78
+/UAS-nebu RNAi HMS01072	52.4 ± 4.7		77.2 ± 3.6		75.1 ± 3.7	
tub-GAL80^{ts}; 54H02-GAL4> UAS-nebu RNAi HMS01072	52.5 ± 4.6		77.5 ± 5.5		79.3 ± 5.1	
tub-GAL80^{ts}; 54H02-GAL4/+	62.0 ± 4.0	n = 10<br>$F_{2,27}$ = 0.06<br>P = 0.95	65.9 ± 4.4	n = 10<br>$F_{2,27}$ = 2.02<br>P = 0.15	72.4 ± 4.1	n = 10<br>$F_{2,27}$ = 0.06<br>P = 0.95
+/UAS-nebu RNAi GD2444	63.5 ± 3.2		72.2 ± 4.1		70.7 ± 4.8	
tub-GAL80^{ts}; 54H02-GAL4> UAS-nebu RNAi GD2444	62.2 ± 3.4		61.1 ±3.1		72.6 ± 4.9	
tub-GAL80^{ts}; alrm-GAL4/+	45.2 ± 4.8	n = 10<br>$F_{2,27}$ = 0.25<br>P = 0.78	51.3 ± 5.1	n = 7<br>$F_{2,18}$ = 0.20<br>P = 0.82	54.0 ± 6.0	n = 7<br>$F_{2,18}$ = 0.26<br>P = 0.77
+/UAS-ALAT RNAi GD9174	41.6 ± 3.1		49.7 ± 4.6		53.7 ± 7.0	
tub-GAL80^{ts}; alrm-GAL4> UAS-ALAT RNAi GD9174	44.7 ± 3.7		54.0 ± 4.8		48.3 ± 5.8	
tub-GAL80^{ts}; alrm-GAL4/+	39.2 ± 6.7	n = 13<br>$F_{2,36}$ = 0.83<br>P = 0.45	64.2 ± 3.5	n = 14<br>$F_{2,39}$ = 0.48<br>P = 0.62	48.5 ± 5.4	n = 12<br>$F_{2,33}$ = 0.40<br>P = 0.67
+/UAS-ALAT RNAi HMC05124	44.1 ± 3.9		63.1 ± 4.0		51.3 ± 4.5	
tub-GAL80^{ts}; alrm-GAL4> UAS-ALAT RNAi HMC05124	48.5 ± 4.5		59.1 ± 4.1		54.0 ± 2.7	

Supplementary Table 7. Efficiency of genetic knockdowns used in the study. Statistical comparisons were done using two-sided unpaired t-test. Asterisks illustrate the significance level, with the following nomenclature: * P<0.05; **P < 0.01; ***P<0.001; ns: not significant, P > 0.05.

Genotypes	Mean ± SEM	Statistics	% of mRNA reduction
elav-GAL4/+	0.35 ± 0.02	n = 4<br>t = 5.75<br>P = 0.001 (**)	75 %
elav-GAL4> UAS-Mpc1 RNAi HMS05634	0.09 ± 0.03		
elav-GAL4/+	0.26 ± 0.02	n = 4<br>t = 3.10<br>P = 0.02 (*)	43 %
elav-GAL4> UAS-Mpc1 RNAi KK102734	0.15 ± 0.02		
elav-GAL4/+	0.15 ± 0.02	n = 4<br>t = 4.15<br>P = 0.006 (**)	60%
elav-GAL4> UAS-ALAT RNAi GD9174	0.059 ± 0.003		
elav-GAL4/+	0.15 ± 0.01	n = 9<br>t = 2.29<br>P = 0.03 (*)	40%
elav-GAL4> UAS-ALAT RNAi HMC05124	0.09 ± 0.01		
+	0.153 ± 0.008	n = 4<br>t = 18.42<br>P = 2.10^{-6} (***)	99%
$CG1640^{EY06928}$	0.0022 ± 0.0003		
repo-GAL4/+	0.024 ± 0.001	n = 4<br>t = 3.74<br>P = 0.009 (**)	26%
repo-GAL4 > UAS-glug RNAi GD2869	0.0177 ± 0.0006		
repo-GAL4/+	0.12 ± 0.01	n = 5<br>t = 2.54<br>P = 0.03 (*)	33%
repo-GAL4 > UAS-nebu RNAi HMS01072	0.08 ± 0.01		
repo-GAL4/+	0.089 ± 0.004	n = 3<br>t = 6.49<br>P = 0.002 (**)	31%
repo-GAL4 > UAS-Treh RNAi HMC03381	0.0613 ± 0.0008		
repo-GAL4/+	N/D		
repo-GAL4 > UAS-Treh RNAi GD5118	lethal		
repo-GAL4/+	0.78 ± 0.04	n = 5<br>t = 2.61<br>P = 0.03 (*)	33%
repo-GAL4 > UAS-GlyP RNAi GD12183	0.52 ± 0.08		
repo-GAL4/+	0.85 ± 0.07	n = 4<br>t = 2.75<br>P = 0.03 (*)	28%
repo-GAL4 > UAS-GlyP RNAi HMS00032	0.61 ± 0.04		
elav-GAL4/+	0.060 ± 0.004	n = 4<br>t = 8.39<br>P = 0.0002 (***)	65%
elav-GAL4 > UAS-hrm RNAi GD1807	0.021± 0.001		

elav-GAL4/+	0.091 ± 0.007	n = 7<br>t = 2.86<br>P = 0.01 (**)	26%
elav-GAL4 > UAS-hrm RNAi HMC03642	0.067 ± 0.002		
elav-GAL4/+	0.17 ± 0.02	n = 4<br>t = 4.13<br>P = 0.006 (**)	64%
elav-GAL4 > UAS-Mct1 RNAi KK108618	0.061 ± 0.009		
repo-GAL4 /+	0.075 ± 0.013	n = 6<br>t = 2.74<br>P = 0.021 (*)	60%
repo-GAL4 > UAS-LDH RNAi HMS00039	0.030 ± 0.010		
repo-GAL4 /+	0.044 ± 0.009	n = 7<br>t = 1.84<br>P = 0.09 (ns)	39%
repo-GAL4 > UAS-LDH RNAi KK102330	0.027 ± 0.003		
UAS-PFK RNAi HMS01324			~80% (ref. [70])
UAS-PFK RNAi KK101887			Lethal with elav-GAL4 (ref. [71])
UAS-PDHE1β RNAi KK107865			~40% (ref. [72])
UAS-Chk RNAi GD1829			~40% (ref. [22])
UAS-Sln RNAi KK104306			~85% (ref. [22])
UAS-Sln RNAi GD1940			~50% (ref. [22])

Supplementary Table 8. Drosophila strains used in this study.

Fly strain	Stock n°/Source/ reference
VT30559-GAL4	VDRC 206077
repo-GAL4	BDSC 7415
alrm-GAL4	[73]
elav-GAL4	[22]
alrm-GAL4; mCD8::GFP	This report
56F03-GAL4	BDSC 39157
54H02-GAL4	[74]
13F02-LexA	BDSC 52460
tub-GAL80^{ts}	BDSC 7019
tub-GAL80^{ts}; VT30559-GAL4	[23]
tub-GAL80^{ts}; repo-GAL4	This report
tub-GAL80^{ts}; alrm-GAL4	[21]
tub-GAL80^{ts}; 56F03-GAL4	[21]
tub-GAL80^{ts}; 54H02-GAL4	[21]
tub-GAL80^{ts}; VT30559-GAL4, UAS-Pyronic	[23]
tub-GAL80^{ts}, 13F02-LexA; 54H02-GAL4	[21]
tub-GAL80^{ts}, 13F02-LexA; alrm-GAL4	This report
tub-GAL80^{ts}; 54H02-GAL4, UAS-FLII12Pglu-700µδ6	[21]
UAS-Mpc1 RNAi HMS05634	BDSC 67817
UAS-Mpc1 RNAi KK102734	VDRC 103829
UAS-PDHE1β RNAi HMC03762	BDSC 55619
UAS-PDHE1β RNAi KK107865	VDRC 104022
UAS-PFK RNAi KK101887	VDRC 105666
UAS-PFK RNAi HMS01324	BDSC 34336
UAS-LDH RNAi KK102330	VDRC 110190
UAS-LDH RNAi HMS00039	BDSC 33640
UAS-PFK RNAi KK101887; UAS-LDH RNAi HMS00039	This report

UAS-ALAT RNAi GD9174	VDRC 32681
UAS-ALAT RNAi HMC05124	BDSC 60130
UAS-PFK RNAi KK101887; UAS-ALAT RNAi GD9174	This report
UAS-glug RNAi GD2869	VDRC 42627
UAS-glug RNAi NIG CG31100	NIG CG31100R-I
UAS-nebu RNAi HMS01072	BDSC 34598
UAS-nebu RNAi GD2444	VDRC 8359
UAS-InR RNAi HMS03166 ; UAS-nebu RNAi HMS01072	This report
UAS-Treh RNAi HMC03381	BDSC 51810
UAS-Treh RNAi GD5118	VDRC 30730
UAS-GlyP RNAi GD12183	VDRC 27928
UAS-GlyP RNAi HMS00032	BDSC 33634
UAS-hrm RNAi GD1807	VDRC 7314
UAS-hrm RNAi HMC03642	BDSC 52902
UAS-Mct1 RNAi KK108618	VDRC 106773
UAS-Chk RNAi GD1829	VDRC 37139
UAS-Sln RNAi KK104306	VDRC 109464
UAS-Sln RNAi GD1940	VDRC 4607
CG1640 EY06928	BDSC 16771
UAS-Pyronic	[23]
UAS-FLII12Pglu-700µδ6	[75]
LexAop-FLII12Pglu-700µδ6	[21]
LexAop-FLII12Pglu-700µδ6; UAS-glug RNAi GD2869	This report
LexAop-FLII12Pglu-700µδ6; UAS-nebu RNAi HMS01072	This report
LexAop-iGlucoSnFR	[67]
LexAop-iGlucoSnFR ; UAS-nebu RNAi HMS01072	This report
LexAop-iGlucoSnFR ; UAS-InR RNAi HMS03166	This report
LexAop-Pyronic; UAS-ALAT RNAi GD9174	This report
LexAop-Pyronic; UAS-PFK RNAi HMS01324	This report

LexAop-Pyronic; UAS-glug RNAi GD2869	This report
LexAop-Pyronic; UAS-nebu RNAi HMS01072	This report
LexAop-Pyronic; UAS-nebu RNAi GD2444	This report
ALAT-HA	This report
nebu-HA	This report
glug-HA	This report

Supplementary Table 9. Primer sequences used for quantitative PCR.

**Target**	**Forward**	**Reverse**
Mpc1	CTCAAAGGAGTGGCGGGATT	TCTTTTGTGTGTCGGCGAGA
ALAT	GGAACAAGCGGCGATTTCATT	CCGCGAACGGCATATTCCA
glug	GCGATGTGGAAGTCA	TGGGGAAGCCCAAGG
nebu	GCGAGGAGGCCAACAAAAAG	GCTCCATTTTCGGCGGATTC
Treh	TGGTCGAGGGCCTAAACAAC	CGCCGCAAAGTTTGTCTTCA
GlyP	TCCACCCTGAGGGACTACTAC	GGTGTTGGTCAGTGAGCGAC
hrm	TCCGACCAAAACGCCATAGC	CGAAGGCTGCCTGGATACC
Ldh	ATACACCTCCTGGGCCATTG	CAATGCCATGTTCGCCCAAA
Mct1	AGGCTACACGGCTTGGATTG	AACATGCTCACGATGATGCAG
Tub	TTGTCGCGTGTGAAA	CTGGACACCAGCCTG

Supplementary video 1
Time course of FRET pyruvate imaging in MB neuron axons (VT30559-GAL4>UAS-Pyronic) upon sodium azide (NaN_3) application (60 s). Top channel: mTFP, bottom channel : Venus.

Supplementary video 2
Time course of FRET glucose imaging in MB neuron cell bodies (13F02-LexA>LexAop- FLII12Pglu-700µδ6) upon validamycin A (ValA) application (100 s). Top channel: YFP, bottom channel : CFP.

Supplementary video 3
Time course of glucose imaging in cortex glia (13F02-LexA>LexAop-iGlucoSnFR) upon validamycin A (ValA) application (100 s).

Additional references

70. Li, H., Hurlburt, A. J. & Tennessen, J. M. A Drosophila model of combined D-2- and L-2-hydroxyglutaric aciduria reveals a mechanism linking mitochondrial citrate export with oncometabolite accumulation. *Dis. Model. Mech.* **11**, dmm035337 (2018).

71. Wu, C.-L. *et al.* Mushroom body glycolysis is required for olfactory memory in Drosophila. *Neurobiol. Learn. Mem.* **150**, 13–19 (2018).

72. Dung, V. M. *et al.* Neuron-specific knockdown of Drosophila PDHB induces reduction of lifespan, deficient locomotive ability, abnormal morphology of motor neuron terminals and photoreceptor axon targeting. *Exp. Cell Res.* **366**, 92–102 (2018).

73. Doherty, J., Logan, M. A., Taşdemir, O. E. & Freeman, M. R. Ensheathing glia function as phagocytes in the adult Drosophila brain. *J. Neurosci.* **29**, 4768–4781 (2009).

74. Coutinho-Budd, J. C., Sheehan, A. E. & Freeman, M. R. The secreted neurotrophin Spätzle 3 promotes glial morphogenesis and supports neuronal survival and function. *Genes Dev.* **31**, 2023–2038 (2017).

75. Takanaga, H., Chaudhuri, B. & Frommer, W. B. GLUT1 and GLUT9 as major contributors to glucose influx in HepG2 cells identified by a high sensitivity intramolecular FRET glucose sensor. *Biochim. Biophys. Acta* **1778**, 1091–1099 (2008).
