# Supplementary material for: Glycolysis-derived alanine from glia fuels neuronal mitochondria for memory in Drosophila
Source: Nat Metab. 2023 Nov 6;5(11):2002–19. doi: 10.1038/s42255-023-00910-y (PMC10663161; doi:10.1038/s42255-023-00910-y)
Supplement: Supplementary file 2 — Reporting Summary [file 42255_2023_910_MOESM2_ESM.pdf]

nature portfolio

Corresponding author(s): Pierre-Yves Plaçais and Thomas Preat

Last updated by author(s): Sep 6, 2023

# Reporting Summary

Nature Portfolio wishes to improve the reproducibility of the work that we publish. This form provides structure for consistency and transparency in reporting. For further information on Nature Portfolio policies, see our Editorial Policies and the Editorial Policy Checklist.

## Statistics

For all statistical analyses, confirm that the following items are present in the figure legend, table legend, main text, or Methods section.

| n/a | Confirmed | |
|---|---|---|
| ☐ | ☒ | The exact sample size (*n*) for each experimental group/condition, given as a discrete number and unit of measurement |
| ☐ | ☒ | A statement on whether measurements were taken from distinct samples or whether the same sample was measured repeatedly |
| ☐ | ☒ | The statistical test(s) used AND whether they are one- or two-sided<br>*Only common tests should be described solely by name; describe more complex techniques in the Methods section.* |
| ☒ | ☐ | A description of all covariates tested |
| ☐ | ☒ | A description of any assumptions or corrections, such as tests of normality and adjustment for multiple comparisons |
| ☐ | ☒ | A full description of the statistical parameters including central tendency (e.g. means) or other basic estimates (e.g. regression coefficient) AND variation (e.g. standard deviation) or associated estimates of uncertainty (e.g. confidence intervals) |
| ☐ | ☒ | For null hypothesis testing, the test statistic (e.g. *F*, *t*, *r*) with confidence intervals, effect sizes, degrees of freedom and *P* value noted<br>*Give P values as exact values whenever suitable.* |
| ☒ | ☐ | For Bayesian analysis, information on the choice of priors and Markov chain Monte Carlo settings |
| ☒ | ☐ | For hierarchical and complex designs, identification of the appropriate level for tests and full reporting of outcomes |
| ☒ | ☐ | Estimates of effect sizes (e.g. Cohen's *d*, Pearson's *r*), indicating how they were calculated |

*Our web collection on statistics for biologists contains articles on many of the points above.*

## Software and code

Policy information about availability of computer code

| | |
|---|---|
| Data collection | In vivo imaging acquisition was performed with the LAS AF Version 2.7.3 software (Leica Microsystems) |
| Data analysis | Statistical analyses were performed with Prism 8 (GraphPad). In vivo imaging data were analyzed using a custom-written MATLAB script previously used in Plaçais et al. Nat. Com. 2017 and de Tredern et al. Cell Rep., 2021 (Mathworks). |

For manuscripts utilizing custom algorithms or software that are central to the research but not yet described in published literature, software must be made available to editors and reviewers. We strongly encourage code deposition in a community repository (e.g. GitHub). See the Nature Portfolio guidelines for submitting code & software for further information.

## Data

Policy information about availability of data

All manuscripts must include a data availability statement. This statement should provide the following information, where applicable:
- Accession codes, unique identifiers, or web links for publicly available datasets
- A description of any restrictions on data availability
- For clinical datasets or third party data, please ensure that the statement adheres to our policy

No datasets that require mandatory deposition into a public database were generated during the current study. Source data that are reported as graphs on figures and extended data figures are available as Supplementary Information alongside the paper. Additional raw data will be shared with no restriction by the corresponding authors upon request.This study made use of the FlyBase database to identify a putative ALAT fly ortholog (http://flybase.org/reports/FBgn0030478),

and of the SCOPE database of single-cell transcriptomics in the fly brain to identify hexose transporters expressed in cortex glia (https://scope.aertslab.org/#/Davie_et_al_Cell_2018/Davie_et_al_Cell_2018%2FAerts_Fly_AdultBrain_Filtered_57k.loom/gene)

## Research involving human participants, their data, or biological material

Policy information about studies with human participants or human data. See also policy information about sex, gender (identity/presentation), and sexual orientation and race, ethnicity and racism.

| | |
|---|---|
| Reporting on sex and gender | No human participant or human data are reported in this study. |
| Reporting on race, ethnicity, or other socially relevant groupings | No human participant or human data are reported in this study. |
| Population characteristics | No human participant or human data are reported in this study. |
| Recruitment | No human participant or human data are reported in this study. |
| Ethics oversight | No human participant or human data are reported in this study. |

Note that full information on the approval of the study protocol must also be provided in the manuscript.

# Field-specific reporting

Please select the one below that is the best fit for your research. If you are not sure, read the appropriate sections before making your selection.

☒ Life sciences ☐ Behavioural & social sciences ☐ Ecological, evolutionary & environmental sciences

For a reference copy of the document with all sections, see nature.com/documents/nr-reporting-summary-flat.pdf

# Life sciences study design

All studies must disclose on these points even when the disclosure is negative.

| | |
|---|---|
| Sample size | No sample size calculation was performed. For both behavior and live imaging experiment, sample size was determined based on previous studies from our group : Plaçais et al. Nat. Com. 2017 and de Tredern et al. Cell Rep., 2021. Sample size for qPCR was determined based on another previous study from our group: Silva et al., Nat. Metabolism 2022. For enzymatic activity measurement, sample size was determined based on pilot experiments comparing wild-type and ALAT mutant line. |
| Data exclusions | No data was excluded in this study |
| Replication | All experiments were repeated in at least 2 independent experiments. In vivo imaging experiments were replicated in at least 4 independent experiments. All replications were successful. |
| Randomization | Flies were assigned to experimental groups based on their genotypes. |
| Blinding | Investigators were not blinded, as for each experiment the same investigator performed crosses, data collection and analysis. |

# Reporting for specific materials, systems and methods

We require information from authors about some types of materials, experimental systems and methods used in many studies. Here, indicate whether each material, system or method listed is relevant to your study. If you are not sure if a list item applies to your research, read the appropriate section before selecting a response.

### Materials & experimental systems

| n/a | Involved in the study | |
|---|---|---|
| ☐ | ☒ | Antibodies |
| ☒ | ☐ | Eukaryotic cell lines |
| ☒ | ☐ | Palaeontology and archaeology |
| ☐ | ☒ | Animals and other organisms |
| ☒ | ☐ | Clinical data |
| ☒ | ☐ | Dual use research of concern |
| ☒ | ☐ | Plants |

### Methods

| n/a | Involved in the study | |
|---|---|---|
| ☒ | ☐ | ChIP-seq |
| ☒ | ☐ | Flow cytometry |
| ☒ | ☐ | MRI-based neuroimaging |

# Antibodies

| | |
|---|---|
| Antibodies used | The following primary antibodies were used: rat anti-HA (1:400; Roche, cat. # 11867423001), mouse anti-nc82 (1:100, DSHB), mouse anti-Wrapper (1:100, DSHB) and rabbit anti-GFP (1:400; Thermo Scientific, A11122).<br>The following secondary antibodies were used: anti-rat conjugated to Alexa Fluor 488 (1:400; Invitrogen, A11006), anti-mouse conjugated to Alexa Fluor 594 (Invitrogen, A11005), anti-rat conjugated to Alexa Fluor 594 (Invitrogen, A11007) and anti-rabbit conjugated to Alexa Fluor 488 (Invitrogen, A11034) |
| Validation | anti-HA: https://antibodyregistry.org/search.php?q=AB_390918<br>anti-nc82: https://antibodyregistry.org/search.php?q=AB_2314866<br>anti-wrapper: https://antibodyregistry.org/search.php?q=AB_528514<br>anti-GFP: https://antibodyregistry.org/search.php?q=AB_221569 |

# Animals and other research organisms

Policy information about studies involving animals; ARRIVE guidelines recommended for reporting animal research, and Sex and Gender in Research

| | |
|---|---|
| Laboratory animals | Experiments involved Drosophila melanogaster. The reference strain was Canton S. All transgenic lines used in the study (reported in Supplementary Table 8) were outcrossed to the reference strain. Experiments were done on 1-4 day-old adult flies. |
| Wild animals | Wild animals were not used in this study |
| Reporting on sex | For behavior experiments, both mated male and female flies were used. However, key results were repeated in separate experiments where memory performance from males and females was assessed separately. Memory phenotypes obtained in males and females were similar. For other experiments (imaging and biochemistry experiments) mated female flies were used because of their larger size. |
| Field-collected samples | No field-collected samples were used in this study |
| Ethics oversight | No ethical approval was required for experiments done on Drosophila melanogaster. |

Note that full information on the approval of the study protocol must also be provided in the manuscript.
